# Exploring Cultural Influences in the Associations Between Emotion Regulation and Mental Health: A Systematic Review Comparing East Asian and Western Cultural Contexts

**DOI:** 10.1002/cpp.70276

**Published:** 2026-04-21

**Authors:** Larissa Shiying Qiu, Marcus Lai, Asami Koike, Martin Sellbom, Belinda J. Liddell, Laura Jobson

**Affiliations:** ^1^ Turner Institute for Brain and Mental Health, School of Psychological Sciences Monash University Clayton Victoria Australia; ^2^ Shapes and Sounds Pty Ltd North Melbourne Victoria Australia; ^3^ School of Psychological Sciences University of Newcastle Callaghan New South Wales Australia

**Keywords:** cognitive appraisal, culture, East Asian, emotion regulation, mental health

## Abstract

Culture shapes how individuals perceive, experience and regulate emotions. Emotion regulation literature, which informs current understandings of emotional disorders, is largely guided by Western frameworks. This review aimed to systematically examine cultural influences on emotion regulation use, and associated mental health outcomes, among individuals from East Asian and Western cultural contexts. The literature search was conducted across five databases in June 2024; the final review included 54 articles. First, findings revealed that East Asian individuals tended to engage more frequently in rumination and avoidance, endorse higher levels of secondary control appraisals (i.e., adjusting oneself to accommodate situational needs) and evaluate the self less favourably than those with Western cultural backgrounds. Second, evidence regarding cultural differences in suppression and reappraisal use was inconclusive. Third, associations between putatively maladaptive regulation strategies (e.g., suppression, avoidance and rumination) and mental health difficulties were less pronounced, absent or at times beneficial among East Asians. Fourth, perceived lack of secondary control was found to have more negative impacts on the mental health of East Asians. Finally, emerging research suggests that the use and effectiveness of emotion regulation strategies need to be considered in relation to individuals' endorsement of specific cultural values. In sum, this review highlights the importance of considering cultural influences on emotional processes, with key implications for clinical practices that target emotion regulation. Future research needs to explore within‐strategy nuances (e.g., regulation for specific emotional valence) and strategies that are informed by East Asian cultural values (e.g., acceptance) to advance cross‐cultural understanding of emotion regulation.

## Summary

- Avoid assuming universality in the use and effectiveness of emotion regulation strategies, and interpret these strategies within their cultural context. Clinicians should critically reflect on whether applying ‘maladaptive’ labels (e.g., suppression, avoidance and rumination) is culturally appropriate before targeting them in treatment. Among East Asian individuals, strategies such as suppression, rumination and avoidance were not consistently associated with poorer mental health and were at times neutral or even beneficial. Assessment and formulation should therefore prioritise the cultural meaning, intention and interpersonal function of these strategies rather than relying solely on Western‐derived assumptions.
- Assess and consider secondary control appraisal. Secondary control (i.e., adjusting oneself to accommodate situational demands) appears particularly salient for East Asian individuals, and perceived lack of secondary control may confer greater psychological risk. Therapeutic work may benefit from strengthening adaptive forms of acceptance, flexibility and context‐sensitive adjustment where culturally congruent.
- Explore clients' cultural values when tailoring interventions. The effectiveness of emotion regulation strategies may depend on individuals' endorsement of specific cultural values (e.g., how interdependent they perceive themselves in relation to social others). Clinicians should assess cultural identity, value orientation and beliefs about adversity and emotion at the individual level, rather than assuming universality of cultural values based on cultural group membership, to understand regulatory goals and guide person‐centred treatment.

Culture shapes how individuals interpret life events and regulate emotions in response to these events. For decades, theoretical accounts, supported by a strong body of empirical evidence, have identified that emotion regulation plays a central role in the development and maintenance of a diverse range of emotional disorders (Lincoln et al. 2022). Consequently, evidence‐based interventions target emotion regulation in improving mental health and well‐being (Gratz et al. 2015). Although research has made extensive progress in our understanding of emotion regulation in psychopathology, the field has progressed with a predominant focus on Western understandings of emotion regulation and little consideration of culture.

The field of psychopathology has often assumed universality in the ways in which emotion regulation underpins emotional disorders. Specifically, the focus has been predominately on Western populations and thus, with emphasis on Western cultural norms, values and belief systems (Jobson 2009), namely, cultural heritage, traditions and beliefs that have evolved primarily from European societies (Eliassen 2023). This is problematic for global mental health and the provision of responsive interventions for culturally and linguistically diverse (CALD) communities. For instance, East Asian cultures (i.e., Chinese, Japanese and Korean), which comprise over 20% of the world's population (Worldometer 2025), share similar core values, traditions and belief systems (e.g., Confucianism, Taoism and Buddhism) originating in ancient Chinese influences that guide morality, behaviour, conduct and social interactions (Lu and Bodur 2011). Although there is heterogeneity within these groupings, significant research has demonstrated Western and East Asian cultures have distinct cultural norms, values and socialisation practices that influence how individuals engage in emotion regulation (e.g., de Vaus et al. 2018). This study aimed, therefore, to systematically review the evidence on cultural differences between East Asian and Western individuals in (a) the use of emotion regulation strategies (i.e., how frequently they are employed) and (b) their effectiveness (i.e., the extent to which these strategies are associated with mental health outcomes across cultural contexts).

## 1 Theories of Emotion Regulation

Emotion regulation is the process by which one attempts to modulate the occurrence, duration and intensity of one or more aspects of emotion (Gross 1998). Difficulties in emotion regulation are a transdiagnostic construct observed in over 75% of psychopathological diagnosis (American Psychiatric Association 2013; Gross and Jazaieri 2014). Consequently, a main focus of emotion regulation research has been on categorising different types of regulation strategies and understanding their associated psychological outcomes (Gross 2015).

Several prominent theories underpin emotion regulation, and it is beyond the scope of this review to outline them all (see Gross 2015; Koole 2009, for reviews). Rather, here, we highlight three influential theories that have informed clinical research. The Transactional Model of Stress (Lazarus and Folkman 1984) and the Modal Model of Emotion (Werner and Gross 2010) both highlight a dual‐process relationship in how individuals differ in reactions to stress. Specifically, when one appraises a situation in a maladaptive way, or as exceeding their ability to cope, this leads to negative emotional responses. Without effective emotion regulation, one may consequently experience challenges to mental health. Gross' (1998) Process Model of Emotion Regulation specifies five broad dimensions of regulatory processes: (1) situation selection (i.e., taking actions to avoid or engage in a situation that may elicit desirable or undesirable emotions, such as situational avoidance), (2) situation modification (i.e., directly changing aspects of a situation to alter its emotional impact, such as problem solving), (3) attentional deployment (i.e., directing or redirecting one's attention to different aspects of the situation, such as distraction, rumination and worry), (4) cognitive change (i.e., changing the way one interprets the situation, such as cognitive reappraisal), and (5) response modulation (i.e., changing one's behaviour or response as a result of the elicited emotion, such as expressive suppression, experiential avoidance and acceptance) (Gross 1998, 2015).

Emotion regulation is deemed maladaptive if it fails to change one's emotional responses in a desirable way or if the detrimental long‐term outcome outweighs the short‐term benefits (Werner and Gross 2010). Concerning mental health, ineffective emotion regulation can occur when a strategy is not appropriately implemented, is inflexible, lacks context‐sensitivity or misaligns with one's long‐term goals (Werner and Gross 2010). For example, suppression is generally regarded as maladaptive due its negative implications on mental health (Dryman and Heimberg 2018; Hu et al. 2014), whereas reappraisal is generally considered an adaptive strategy associated with better mental health and quality of life (Dryman and Heimberg 2018; Hu et al. 2014).

Cognitive appraisal refers to an evaluative process that rests on an individual's subjective interpretation of a specific situation or transaction (Lazarus and Folkman 1984). Cognitive appraisal plays a key role in how individuals regulate emotions because the way an event is appraised determines emotional responses. The appraisal process takes place as individuals make sense of their experiences in accordance with its cultural meanings (Mesquita and Albert 2007). Hence, in this systematic review, we have included appraisal regulation as a key emotion regulation strategy, particularly given its strong influence on the aetiology and maintenance of emotional disorders.

## 2 Culture Influences Emotion Regulation and Its Functionality

Theoretical models of emotion regulation acknowledge that the effectiveness of an emotion regulation strategy needs to be considered in its cultural context (Gross 2015; Lazarus and Folkman 1984). Culture shapes the way an individual thinks, feels and behaves (Ford and Mauss 2015). East Asian cultures are rooted in Taoism, Buddhism and Confucianism philosophies, providing valuable insights into unique societal belief systems that guide individuals' interpretations of emotions and emotional experiences, regulatory goals and preferred regulation strategies (Lai et al. 2025; Lin et al. 2021; Xie and Wong 2021). For instance, Taoism emphasises nature, which signifies the importance of not forcing one's way through life and learning to accept, tolerate and embrace contradictions (Lin et al. 2021). Similarly, Buddhist views focus on the interconnectedness of all beings, encouraging individuals to acknowledge the impermanence and interdependence of all phenomena, and adopt a nonjudgmental awareness of the present moment (Lin et al. 2021). Confucianism emphasises aspiring to become the best possible self to ensure community and societal benefits (Lin et al. 2021).

These three philosophies foster a worldview wherein members of East Asian cultures approach emotions through a holistic lens, emphasising the understanding of emotions within their broader contexts (de Vaus et al. 2018; Ishii and Eisen 2021). As a result, positive and negative emotions are perceived as co‐occurring rather than opposing states. Negative emotions (e.g., shame and guilt) are viewed as valuable, offering opportunities for self‐reflection and personal growth, whereas an excess of positive emotions (e.g., happy and proud) may be potentially disruptive or leading to undesirable outcomes (e.g., jealousy and inappropriate emphasis on autonomy; de Vaus et al. 2018; Ishii and Eisen 2021; Mesquita and Albert 2007). This perspective contrasts with Western analytic views, which often view negative emotions as undesirable and as opposite to positive emotions, subsequently emphasising the need to downregulate negative emotions or upregulate positive emotions (de Vaus et al. 2018; Gross 2015). This approach to emotions results in cultural differences in regulatory goals and the selection of strategies, which enables East Asians to approach negative emotions with greater acceptance and curiosity, fostering greater flexibility in using emotion regulation strategies that are responsive to specific contextual demands (Chen et al. 2024; de Vaus et al. 2018).

Similarly, Confucianism virtues, which emphasise social relationships within relational hierarchy, have shaped a collective perception of the self in relation to others among East Asian societies (Lin et al. 2021). This cultural emphasis on interdependence tends to view the self as interconnected with others and prioritises collective wellbeing and interpersonal harmony over individual achievements (Markus and Kitayama 1991). This in turn shapes one's regulatory goals. Emotion regulation is often guided by the expectations of others and one's social roles, with the goal to maintain harmony, fulfil relational obligations and preserve group cohesion (Markus and Kitayama 1991). Emotions are not simply managed for individual relief or self‐expression but are regulated in ways that minimise social burden and disruption, often through restraining expression or internal adaptation strategies (e.g., acceptance, appraisals and avoidance) that align the self with contextual demands (Mesquita and Albert 2007). Further, as relationships can be perceived as a source of strength (Xie and Wong 2021), East Asian individuals are more willing to engage in and receive benefits from interpersonal emotion regulation strategies, through processes, such as social modelling and perspective taking (Ishii and Eisen 2021; Liddell and Williams 2019). This contrasts with the independent values promoted by many Western individualistic cultures, where regulatory goals centre around affirming autonomy, asserting individuality, expressing the authentic self and enhancing personal well‐being (Markus and Kitayama 1991). Although these philosophies provide valuable insights into societal belief systems that shape interpretations of emotions, regulatory goals and preferred regulation strategies, it is important to acknowledge that such cultural groupings are neither monolithic nor universally representative of all individuals within them.

Adaptive emotion regulation requires alignment between regulatory processes and a person's broader priorities set by their sociocultural context, such as values, goals, beliefs and preferred regulatory styles (Lazarus and Folkman 1984). Regulation that contributes to emotions that are congruent with cultural norms has positive implications for wellbeing (Ishii and Eisen 2021). Conversely, when the regulatory process conflicts with these priorities, it can become a new source of stress and contribute to psychopathology (Ford and Mauss 2015). Similarly, when implemented strategies are incongruent with an individual's goals, cultural values and norms, they may become a new source of stress or be reluctantly used and applied with limited confidence, consequently reducing their effectiveness (Lazarus and Folkman 1984; Mesquita and Albert 2007).

## 3 Current Study

The aim of this systematic review was to evaluate the current understanding of cultural influences, specifically, East Asian compared to Western, on the use and contextual effectiveness of emotion regulation and their associated mental health outcomes. To address this aim, we proposed two research questions: (1) In what ways does the use of emotion regulation strategies vary across East Asian and Western cultural contexts, and (2) how does culture influence the association between emotion regulation strategies and mental health outcomes (i.e., contextual effectiveness of strategies)? Although cross‐cultural studies tend to compare members from Eastern and Western cultural group, emerging cross‐cultural theories signified the importance of also considering the extent to which an individual is oriented towards a particular cultural value (e.g., independent or interdependent self‐construal), as they drive the motive for regulation and the effectiveness of a given strategy (Ford and Mauss 2015). Hence, in this review, we considered culture as not solely one's cultural group but also their endorsement of relevant cultural values.

## 1 Method

### 1.1 Research Team

Our review team included researchers with a diverse range of cultural, clinical and research backgrounds. We recognise that this diversity of perspectives influences our interpretations and conclusions. The research was led by East Asian clinical researchers and supported by researchers with experience in mental health and/or cross‐cultural psychology.

### 1.2 Literature Search

This systematic review was preregistered with Prospero (CRD42024536519). It was conducted following the Preferred Reporting Items for Systematic Review and Meta‐Analysis (PRISMA) guidelines. Relevant MeSH headings and free text words were used for titles and abstracts search across PsycINFO, MEDLINE, Scopus, PTSDpubs and Social Science Database. The search strategy and database selection were developed in consultation with a university librarian to ensure comprehensive coverage of all relevant domains of interest. The search occurred in June 2024.

Based on our research questions, the literature search and inclusion criteria were informed by three main themes: (1) cross‐cultural comparisons; (2) emotion regulation, including but not limited to suppression, cognitive appraisals and reappraisal, acceptance, avoidance, rumination, worry, distraction, problem‐solving, interpersonal emotion regulation and difficulties in emotion regulation; and (3) mental health (including wellbeing). We chose not to include specific mental health outcome search terms (e.g., depression and life satisfaction), as such terms are often based on Western conceptualisations of psychopathology and wellbeing and do not allow for cross‐cultural complexities. The complete list of search terms can be found in Table S1.

### 1.3 Inclusion and Exclusion Criteria

Inclusion criteria were articles that (1) focused on at least one emotion regulation strategy, where emotion regulation was operationalised as a strategy used to modify the occurrence, intensity or duration of emotional responses (Gross 1998); (2) direct comparison of participants from at least one East Asian cultural group (Japan, Korea, Mainland China, Hong Kong, Macau and Taiwan^1^) with one Western cultural group; (3) examined cultural influences on the association between emotion regulation and mental health; (4) included participants aged 18 or above; and (5) were empirical studies.

Articles were excluded if they (1) were single case studies or had no original data (e.g., systematic review and meta‐analysis), (2) only investigated one cultural group without including a comparison cultural group and (3) lacked analyses that examined cultural influence on the relationship between emotion regulation and mental health. There was no restriction on articles with original data; cross‐sectional, longitudinal, qualitative, quasi‐experimental studies and randomised control trials of both clinical and community samples were included. We did not attempt to exclude non‐English papers due to the cross‐cultural nature of this review, although all eligible articles were published in English.

### 1.4 Screening and Included Studies

**FIGURE 1:**
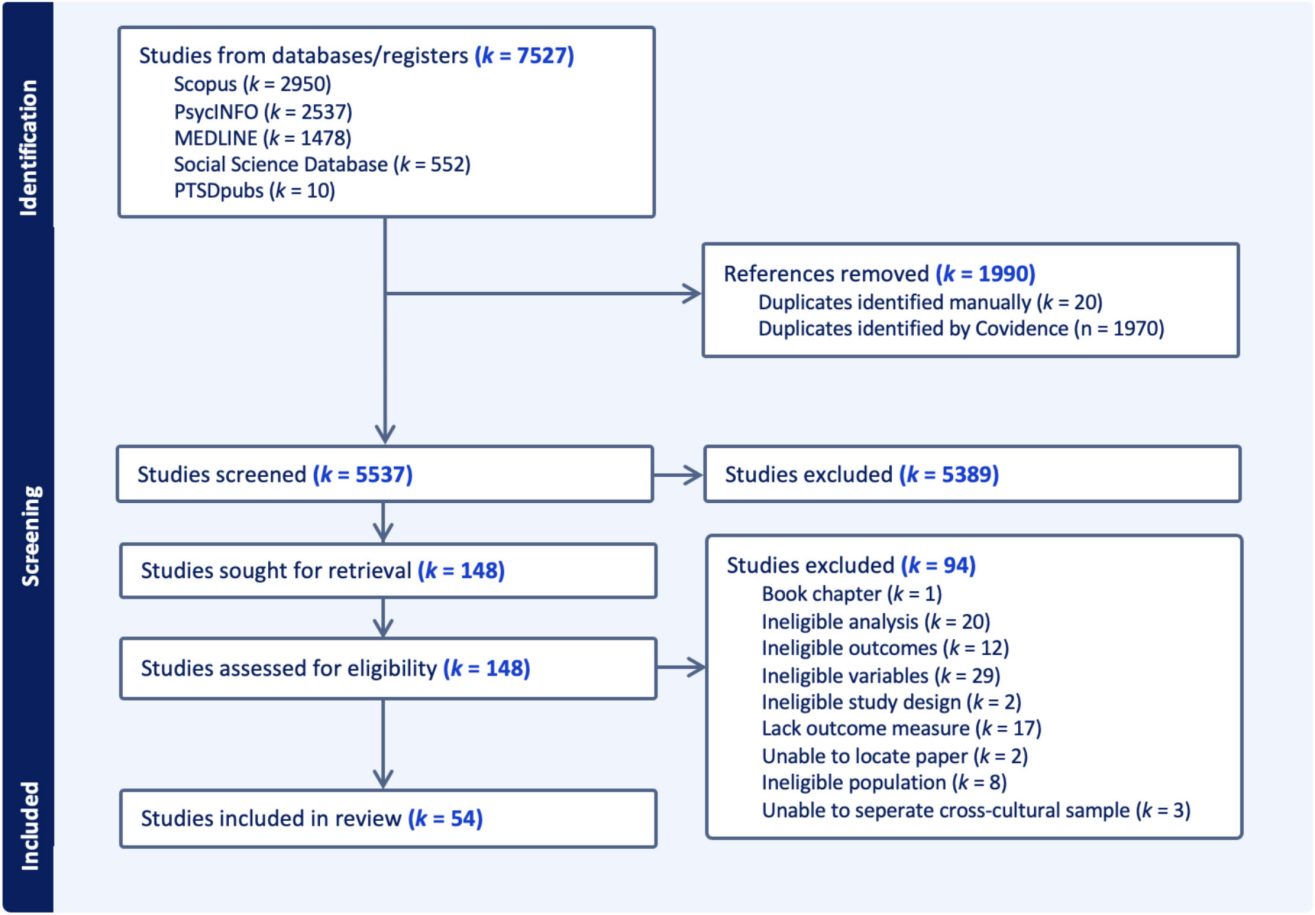
PRISMA flowchart.

Search outputs returned a total of 7527 articles, which were uploaded to Covidence Reference Management Software. Duplicates were removed by Covidence (*k* = 1970) and by reviewers manually (*k* = 20), which resulted in a total of 5537 articles. Initial title and abstract screening was conducted independently by the first (L.S.Q.) and second (M.L.) authors adhering to the Cochrane Collaboration Guidelines. Subsequently, 148 articles were screened in full texts following the same protocol. A third independent reviewer (L.J.) was consulted to resolve conflicts that arose due to the discrepancies between L.S.Q. and M.L. This resulted in 54 eligible studies that were included for data extraction. More information can be found in the PRISMA flowchart (Figure 1).

### 1.5 Effect Size Calculation

We used *r* as the primary effect size. Standardised beta coefficients were used directly as a proxy for *r* when derived from a simple regression. For multiple regression, beta was converted into *r* following Peterson and Brown's (2005) approach (*r* = *β* + 0.05 * *λ*). When only unstandardised coefficient was reported, it was converted to standardised coefficient (*β* = *B* * (*SD*x / *SD*y)) (Cohen et al. 2003) and in simple regressions, treated *β* as *r*. In cases where *d* was used as primary effect size, conversion was applied using the method outlined by Rosenthal (1994) (*r* ≈ *d* / √(*d* ^2^ + 4)); partial eta squared was converted to *r* following *r* = √ηp^2^ (Cohen 1988). Effect size was considered small if *r* = 0.10, medium for *r* = 0.30 and large if *r* = 0.50 (Cohen 1992).

### 1.6 Quality Assessment

A quality assessment tool was developed to evaluate studies included in this review. Thirteen assessment questions were taken from the Joanna Briggs Institute appraisal tool (Downes et al. 2016; Moola et al. 2020; Ross et al. 2011). Given that cross‐cultural comparison was a key inclusion criterion, and hence central to assessing the quality of included papers, two additional questions were developed by the current research team: (a) Did the study adapt their procedure to improve cultural responsiveness, and (b) did the study include measurement invariance of the scales used in the research? Therefore, the assessment tool contained 15 items; each item was rated based on whether the study methodology fulfiled the criterion specified (1 = yes; 0 = no/unclear), with a higher percentage indicating better research quality whereby more studies met the specific criteria. L.S.Q. and M.L. independently reviewed the quality of each study during data extraction. All conflicts were resolved through discussion and consultation with a third reviewer (L.J.).

## 2 Results

### 2.1 Research Quality

On average, the included studies met 72.83% (SD = 1.71) of the specified quality assessment criteria, suggesting a moderate to high level of research quality. However, several limitations were identified: only 40% (*SD* = 0.49) of studies assessed measurement invariance, representing a concern given the direct cross‐cultural comparison focus of the included articles; many studies reported unclear sampling criteria (36%, SD = 0.48); there was often a lack of power analysis (25%, SD = 0.43) and insufficient justification for missing data (25%, SD = 0.43). Unclear information on ethical approval and participation consent (64%, SD = 0.48) was more commonly found in studies that were dated (e.g., study from 1989 and early 2000) and among dissertations. Mean and standard deviations for each criteria can be found in Table S2.

### 2.2 Study and Sampling Characteristics

Information on study designs and sample characteristics can be found in Table S3. The review included 54 articles, amounting to 54 unique samples—noting that one study included two unique samples (Haliczer et al. 2020)—whereas two studies shared the same sample (Akutsu et al. 2016; Yamaguchi et al. 2015). Over half of the studies (*k* = 31, 57.4%) recruited university students, with 12 studies (22.2%) recruiting from the general community, two studies (3.7%) sampling both university students and the general community, two studies (3.7%) using caregivers of patients with dementia and two studies (3.7%) recruiting a geriatric sample. The remaining studies (*k* = 5, 9.3%) examined specific groups: college students and young adults from a church community, pregnant women, university employees, women with breast cancer and bereaved parents. Most studies (85.2%, *k* = 46) employed a cross‐sectional design. Two studies (3.7%) utilised a longitudinal design, four studies (7.4%) employed a quasi‐experimental approach, one study (1.9%) employed a qualitative design and one study (1.9%) used mixed‐methods.

### 2.3 Types of Emotion Regulation

A broad range of emotion regulation strategies were examined in the included studies. These included suppression (i.e., expressive suppression, emotional expression, distress disclosure and anger regulation; *k* = 18), reappraisal (i.e., positive thinking and reframing; *k* = 15), acceptance (*k* = 5), interpersonal emotion regulation (i.e., seeking social support to regulate emotions and experimentally induced interpersonal regulation; *k* = 7), avoidance (i.e., escape avoidance, avoidant coping, experiential avoidance and specific deliberate grief avoidance; *k* = 13), problem‐solving (i.e., active coping, confrontive coping and rational problem‐solving; *k* = 8), rumination (i.e., intrusive rumination, deliberate rumination, habitual rumination, trauma‐related rumination, brooding and reflection; *k* = 8), distraction (*k* = 2) and worry (*k* = 1).

Studies focusing on cognitive appraisals included a focus on control appraisals (*k* = 12), broadly distinguished as (a) primary control (i.e., one's perceived emotion regulation capacities and ability to handle difficult situations), (b) secondary control (i.e., control through internal adaptation, fatalistic appraisals and culturally specific beliefs about adversity) and (c) acceptance and the values of adaptiveness. Several studies also focused on other types of cognitive appraisals, including those relating to cultural norms and value judgements (i.e., traditional values, social axioms and self‐evaluation; *k* = 5), goal‐related appraisals (i.e., evaluations of whether events align with one's goals; *k* = 4) and current and future‐oriented expectations (*k* = 4). Detailed findings on cultural differences in emotion regulation use are presented in Table S3.

### 2.4 Outcome Types

As our search strategy did not specify particular mental health constructs, a range of outcomes were captured. Among these, depression emerged as the most commonly examined outcome (*k* = 25), followed by life satisfaction (*k* = 11), distress (*k* = 10), PTSD and trauma‐related outcomes (*k* = 6), anxiety (*k* = 5), affect (*k* = 5), loneliness (*k* = 3), subjective wellbeing (*k* = 4), self‐esteem (*k* = 2) and other mental health outcomes (one study each; hopelessness, happiness, psychological health, borderline personality disorder, psychological strain, obsessive‐compulsive symptoms, paranoid ideation, quality of life and prolonged grief disorder symptoms). Two studies focused on physiological measures of emotional experiences. Detailed findings on cultural differences in the associations between emotion regulation and mental health can be found in Table S4.

### 2.5 Cultural Groups

The review included 116 sample groups. The East Asian Group (59 groups of participants) included 30 samples of Chinese participants (e.g., Hong Kong Chinese [HKC], Taiwanese Chinese and Chinese Americans), 15 samples of Japanese participants, 5 samples of Korean participants and 9 samples of unspecified ‘East Asians’. The most commonly used comparison group was American participants (*k* = 30), followed by German (*k* = 5), European/Caucasian Australian (*k* = 5), Canadian (*k* = 4) and British (*k* = 3). Other Western comparison groups included Swedish, Finnish, Danish and unspecified ‘White’ participants (*k* = 10). Thirty‐seven studies sampled East Asian participants from their country of origin, 14 studies sampled East Asians in Western countries, 2 studies sampled both, and 1 study did not specify. The sampling criteria of culture was most commonly defined by country of residence or geographical location (*k* = 28), ethnicity (*k* = 13), self‐identified cultural identity (*k* = 6), nationality (*k* = 4), ancestry (*k* = 2) and racial identity (*k* = 1); some studies also included inclusion criteria such as mother tongue (*k* = 1) and birthplace (*k* = 1). It is worth noting that most Western samples included participants from diverse ethnic backgrounds.

Six studies included measures of cultural values in the analyses of interest, including self‐construal (Akutsu et al. 2016; Corey and Allen 2013; Jobson et al. 2024; Kalibatseva and Leong 2018; Schunk, Trommsdorff, and König‐Teshnizi 2022), level of holistic and analytic thinking (Jobson et al. 2024) and endorsement of harmony seeking and rejection avoidance (Schunk, Wong, et al. 2022).

### 2.6 Emotion Regulation Findings

#### 2.6.1 Suppression

##### 2.6.1.1 Cultural Differences in Suppression Use

Thirteen studies examined cultural differences in the use of suppression, with eight studies finding no significant cultural group differences, two studies finding greater expressive suppression use among East Asians than Westerners (Hirano and Ishii 2024; Soto et al. 2011) and two studies finding Koreans expressed emotions more than Americans (Aldwin and Greenberger 1987; Kwon et al. 2013). One study found differences depended on emotional valence: Japanese individuals were more likely to suppress positive emotions out of empathetic concern, whereas Westerners reported greater empathetic suppression of negative emotions (Schunk, Trommsdorff, and König‐Teshnizi 2022).

Four studies examined the role of cultural values in shaping the use of suppression (Akutsu et al. 2016; Kalibatseva and Leong 2018; Schunk, Wong, et al. 2022; Tse 2017). Findings revealed those with higher independence tended to suppress less and express emotions more (Akutsu et al. 2016; Kalibatseva and Leong 2018; Tse 2017). However, Kalibatseva and Leong (2018) found independence was linked to less expressive suppression only among Chinese Americans, whereas Akutsu et al. (2016) found independence was only associated with less suppression of anger among Westerners. Tse (2017) found interdependence was associated with greater emotional expression across cultural groups, whereas Akutsu et al. (2016) found it to be associated with less anger expression but not related to anger suppression. Similarly, Kalibatseva and Leong (2018) found no association between interdependence and expressive suppression. Lastly, Schunk, Wong, et al. (2022) found higher levels of harmony seeking were associated with less suppression, whereas higher rejection avoidance correlated with more frequent suppression only among Germans, but not their East Asian counterparts.

##### 2.6.1.2 Cultural Influences on the Associations Between Suppression and Mental Health

Eighteen studies investigated cultural influences on the association between suppression and mental health. Seven studies found suppression was associated with poorer mental health (depression, Kalibatseva and Leong 2018; Kwon et al. 2013; Smith et al. 2016; social anxiety and perceived stress, Yamaguchi et al. 2015; loneliness, Hirano and Ishii 2024; decreased life satisfaction, Kwon and Kim 2019; Smith et al. 2016) regardless of cultural background (East Asian *r* = 0.11–0.36; Western *r* = 0.09–0.59). Notably, this association did at times vary depending on the valence of emotions. Yu et al. (2023) found the suppression of positive, but not negative emotions, to be consistently linked to poorer mental health across cultural groups. Chen et al. (2020) also highlighted that the negative impact of suppression may depend on the type and quality of suppression, with suppression ability, rather than frequency, being associated with fewer depressive symptoms across cultures.

Eight studies indicated suppression was linked to poorer mental health among Westerners but showed significantly attenuated or absent associations among East Asians (depression, Chen et al. 2020; Schunk, Trommsdorff, and König‐Teshnizi 2022; Soto et al. 2011; PTSD, Nagulendran and Jobson 2020; wellbeing, Kwon et al. 2013; Schunk et al. 2021; Schunk, Trommsdorff, and König‐Teshnizi 2022; Schunk, Wong, et al. 2022; Smith et al. 2016; Soto et al. 2011) (East Asian *r* ≤ 0.001–0.36; Western *r* = 0.22–0.74). Some studies also observed unique benefits of suppression for East Asians: for instance, increased parasympathetic activity during stress (Nagulendran et al. 2020) and positive associations between empathic suppression of negative emotions and well‐being, and negative association with depression (Schunk, Trommsdorff, and König‐Teshnizi 2022) (*r* = 0.13–0.35). Notably, emotional expression was at times uniquely linked to negative outcomes for East Asians, including higher depressive (*r =* 0.12; Aldwin and Greenberger 1987) and somatic symptoms (*r =* 0.11; Tse 2017). Additionally, uncontrolled expression of emotions was more harmful for Japanese participants compared to Westerners (Schunk, Trommsdorff, and König‐Teshnizi 2022). Comparatively, Kahn et al. (2017) found that distress disclosure was beneficial for the mental health (i.e., lower depression and higher life satisfaction) of both Taiwanese and Americans. Four studies that explored negative emotions further diverged from the above patterns. Akutsu et al. (2016) found anger suppression was associated with lower life satisfaction among Japanese (*r* = 0.47) than American participants (*r* = 0.33). Anger control and suppression of negative emotions were more strongly linked to positive wellbeing for Westerners but not East Asians (Smith et al. 2016; Yamaguchi et al. 2015; Yu et al. 2023).

##### 2.6.1.3 Suppression Summary

In sum, suppression was associated with poorer mental health, with some evidence that this association may be stronger among Westerners than East Asians. The associations between suppression and mental health may vary depending on emotional valence (e.g., positive vs. negative emotions), type (e.g., frequency vs. ability) and quality (e.g., controllable vs. uncontrollable) of suppression.

#### 2.6.2 Reappraisal

##### 2.6.2.1 Cultural Differences in Reappraisal Use

Ten studies examined cultural differences in the use of reappraisal. Whereas six studies (all of which focused on university students) found no cultural group difference, four studies using community samples reported higher use of reappraisal among Westerners than East Asians (Chataway and Berry 1989; Hirano and Ishii 2024; Schunk, Trommsdorff, and König‐Teshnizi 2022; Schunk, Wong, et al. 2022).

Specific to cultural values, Kalibatseva and Leong (2018) found that regardless of cultural background, independent and interdependent self‐construals were positively associated with cognitive reappraisal. Schunk, Wong, et al. (2022) found that harmony seeking was correlated with higher reappraisal across cultures whereas rejection avoidance was associated with less reappraisal among Germans and HKC but showed no relevance to reappraisal among Japanese.

##### 2.6.2.2 Cultural Influences on the Associations Between Reappraisal and Mental Health

Eleven studies examined the moderating role of culture on the association between reappraisal and mental health. Six studies showed culture did not influence the overall association between reappraisal and mental health. Across cultures, reappraisal was generally positively associated with wellbeing and negatively associated with mental health difficulties (Chung et al. 2020; Hirano and Ishii 2024; Kalibatseva and Leong 2018; Schunk et al. 2021; Schunk, Trommsdorff, and König‐Teshnizi 2022; Schunk, Wong, et al. 2022) (East Asian *r* = 0.15–0.57; Western *r* = 0.18–0.54). Chataway and Berry (1989) found reappraisal was uncorrelated with stress.

However, Schunk, Trommsdorff, and König‐Teshnizi (2022) suggested the association between reappraisal of negative emotions and higher wellbeing/lower depression was stronger among Germans than Japanese participants. Similarly, a quasi‐experiment found that ‘White’ participants derived greater benefits from reappraisal than their East Asian counterparts (He et al. 2021). Shim et al. (2006) found reappraisal was only linked to better quality of life among Germans but not their Japanese and Korean counterparts.

In contrast, Schunk, Wong, et al. (2022) found that the positive effect of reappraisal on life satisfaction was stronger among Japanese than Germans but also found this association to be negative for HKC, indicating nuances within members of different East Asian societies. One study found reappraisal was associated with less depressive symptoms for East Asians only (Kwon et al. 2013), whereas Nagulendran and Jobson (2020) found reappraisal did not differ among East Asians with or without PTSD, whereas Caucasians with PTSD had significantly lower levels of reappraisal than those without.

##### 2.6.2.3 Reappraisal Summary

In sum, there was no clear evidence for cultural differences in the use of reappraisal; reappraisal was generally associated with positive mental health, though this positive association may be stronger among Westerners, but the results remain inconclusive at this stage.

#### 2.6.3 Acceptance

##### 2.6.3.1 Cultural Differences in Acceptance Use

Five studies examined cultural differences in acceptance and results were mixed. One study found no cultural differences in the use of acceptance (Aldwin and Greenberger 1987). A qualitative study found both Chinese and Swedish older adults used acceptance to cope with existential loneliness (Chung et al. 2020). Three studies found cultural differences in the prioritisation and use of acceptance, with two studies finding Westerners prioritised and engaged in more frequent use of acceptance than East Asians (Morling et al. 2003; Schunk, Trommsdorff, and König‐Teshnizi 2022) and one study found East Asians had less emotional nonacceptance than their Western counterparts (Haliczer et al. 2020). In contrast, Turner (2022) found acceptance was a unique narrative theme of Japanese people's identity (as opposed to their American and Danish counterparts).

##### 2.6.3.2 Cultural Influences on the Associations Between Acceptance and Mental Health

Results were inconsistent regarding the role of culture in moderating the association between acceptance and mental health. Two studies found acceptance was linked to poorer outcomes for East Asians (Aldwin and Greenberger 1987; Morling et al. 2003). Haliczer et al. (2020) found no association between nonacceptance and affective instability among East Asians, whereas for Westerners, a positive association was observed. In contrast, Schunk, Trommsdorff, and König‐Teshnizi (2022) reported a nonsignificant but positive association between acceptance and wellbeing for Japanese participants but a nonsignificant negative association for Germans.

##### 2.6.3.3 Acceptance Summary

The results were mixed regarding cultural differences in the use and effectiveness of acceptance.

#### 2.6.4 Interpersonal Emotion Regulation

##### 2.6.4.1 Cultural Differences in Interpersonal Emotion Regulation Use

Seven studies examined cultural differences in interpersonal emotion regulation. Three studies found East Asians prioritised and exercised more interpersonal emotion regulation than their Western counterparts (Adams 2003; Liddell and Williams 2019; Morling et al. 2003), whereas three studies found no cultural differences (Aldwin and Greenberger 1987; Bjorck et al. 2001; Chataway and Berry 1989). One qualitative study reported communal growth as a culturally specific narrative theme central to Danish but not Japanese or American people's identity (Turner 2022).

##### 2.6.4.2 Cultural Influences on the Associations Between Interpersonal Emotion Regulation and Mental Health

Four studies examined the effectiveness of interpersonal emotion regulation strategies across cultures. Across three studies, interpersonal emotion regulation was not cross‐sectionally nor longitudinally associated with distress in either group (Adams 2003; Chataway and Berry 1989; Morling et al. 2003). However, one quasi‐experimental study (Liddell and Williams 2019) found East Asians physiologically benefited more from interpersonal priming than Western Europeans, although these effects did not extend beyond the immediate prime exposure. The same study also found that East Asians were more effective in applying interpersonal strategies to regulate physiological responses to negative cues.

##### 2.6.4.3 Interpersonal Emotion Regulation Summary

The results were mixed regarding cultural differences in the use of interpersonal emotion regulation; recent preliminary evidence suggests East Asian individuals may benefit more from interpersonal emotion regulation regarding psychological outcomes.

#### 2.6.5 Avoidance

##### 2.6.5.1 Cultural Differences in Avoidance Use

Eleven studies examined cultural differences in the use of avoidance. Seven studies found East Asians reported greater use of avoidance than Westerners (Bjorck et al. 2001; Bonanno et al. 2005; Chang and Yang 2024; Hirano and Ishii 2024; Leung et al. 2011; Nishiguchi et al. 2022; Shaffer et al. 2000). Three studies reported no cultural group differences (Adams 2003; Hamamura and Mearns 2019; Perera and Chang 2015). In contrast, Ogawa (2009) found Americans reported more avoidance‐related coping than Japanese but also found cultural nuances related to self‐construal, where avoidance was correlated with higher level of interdependence and lower level of independence across groups.

##### 2.6.5.2 Cultural Influences on the Associations Between Avoidance and Mental Health

The 13 studies examining the moderating effect of cultural group on the relationship between avoidance and mental health outcomes found notable cultural differences. Across seven studies, avoidance was consistently associated with poorer mental health (depression, PTSD, anxiety, obsession‐compulsion, paranoia, hopelessness, loneliness, distress and lower happiness) for individuals from both East Asian and Western cultures (Bjorck et al. 2001; Chang and Yang 2024; Hirano and Ishii 2024; Leung et al. 2011; Nagulendran and Jobson 2020; Perera and Chang 2015; Nishiguchi et al. 2022) (*k* = 6; East Asians *r* = 0.11–0.53; Western *r* = 0.26–0.74). However, the strength of this relationship varied across cultures. One study found that the positive association between avoidance and poor mental health was stronger among Japanese Americans than Anglo Americans (Adams 2003). Contrastingly, five studies (cross‐sectional and longitudinal) found the association between avoidance and negative mental health outcomes to be weaker (Nagulendran and Jobson 2020; Nishiguchi et al. 2022) (*k* = 2, East Asian *r* = 0.17–0.35, Western *r* = 0.26–0.74) or nonsignificant (Bonanno et al. 2005; Hamamura and Mearns 2019; Ogawa 2009) (*k* = 3, East Asian *r* = 0.03–0.13; Western *r* = 0.24–0.49) among East Asians. Notably, two studies also reported no significant association between avoidance and wellbeing across cultures (Shaffer et al. 2000; Shim et al. 2006).

##### 2.6.5.3 Avoidance Summary

East Asians tended to report greater use of avoidance than Westerners. Avoidance was consistently associated with poorer mental health for individuals from both East Asian and Western cultures, with some evidence suggesting this relationship was weaker among East Asian participants.

#### 2.6.6 Problem‐Solving

##### 2.6.6.1 Cultural Differences in Problem‐Solving Use

Eight studies compared cultural groups in the use of problem‐solving. Six studies reported no cultural differences (Adams 2003; Aldwin and Greenberger 1987; Bjorck et al. 2001; Chataway and Berry 1989; Shaffer et al. 2000; Shaw et al. 1997). One study reported that Caucasian Americans used greater active coping strategies than Taiwanese, with independent self‐construal associated with greater problem‐solving (Corey and Allen 2013). Contrarily, one study found Chinese reported higher rational problem‐solving than Americans (Chang and Yang 2024).

##### 2.6.6.2 Cultural Influences on the Associations Between Problem‐Solving and Mental Health

Four studies found no significant association between problem‐solving and distress/stress across cultural groups (Adams 2003; Chataway and Berry 1989; Corey and Allen 2013; Shaffer et al. 2000). Two studies found problem‐solving was uniquely associated with greater depression among East Asians (Aldwin and Greenberger 1987; Chang and Yang 2024) but was protective against loneliness and hopelessness in both Americans and Chinese participants (Chang and Yang 2024). In contrast, Shaw et al. (1997) found behavioural confronting was linked to higher depression and anxiety in Americans but was negatively associated with depression and unrelated to anxiety in Chinese individuals.

##### 2.6.6.3 Problem‐Solving Summary

Most studies found no cultural differences in the use of problem‐solving. Several studies found no association between problem‐solving and psychological outcomes, with other studies highlighting mixed and inconclusive findings regarding cultural differences in this association.

#### 2.6.7 Rumination

##### 2.6.7.1 Cultural Differences in Rumination Use

Five studies compared the use of rumination, with three studies finding East Asians ruminate more than Westerners (Choi and Miyamoto 2023; Kwon et al. 2013; Schunk, Trommsdorff, and König‐Teshnizi 2022) and two studies finding no cultural differences (Schunk, Wong, et al. 2022; Toussaint et al. 2023). One study found rejection avoidance was correlated with greater rumination across cultures, and harmony seeking was unrelated to rumination among Germans but correlated with less rumination among HKC and greater rumination among Japanese (Schunk, Wong, et al. 2022).

##### 2.6.7.2 Cultural Influences on the Associations Between Rumination and Mental Health

Across eight studies, there was consistent evidence demonstrating rumination as maladaptive for mental health (i.e., depression, PTSD, lower life satisfaction and poorer wellbeing) across both East Asian and Western groups (Choi and Miyamoto 2023; Kwon et al. 2013; Nagulendran and Jobson 2020; Schunk et al. 2021; Schunk, Trommsdorff, and König‐Teshnizi 2022; Schunk, Wong, et al. 2022; Toussaint et al. 2023), though the strength of these associations tended to be weaker among East Asian participants (Choi and Miyamoto 2023; Schunk, Trommsdorff, and König‐Teshnizi 2022; Toussaint et al. 2023) (*k* = 7, East Asian *r* = 0.23–0.46, Western *r* = 0.31–0.69). Taku et al. (2009) found cultural differences in rumination and posttraumatic growth: In Americans, recent deliberate rumination was more important than immediate postevent rumination, whereas in Japanese participants, both early and recent deliberate rumination were positively related to posttraumatic growth.

##### 2.6.7.3 Rumination Summary

There was some evidence that East Asians use rumination more than Western participants; rumination was consistently associated with poorer mental health, with some evidence that the strength of these associations tended to be weaker among East Asian participants.

### 2.7 Other Emotion Regulation Strategies

Two studies examined distraction, with one study finding Japanese participants reported greater use of distraction than German participants (Schunk, Trommsdorff, and König‐Teshnizi 2022) and another study finding no cultural differences (Aldwin and Greenberger 1987). One study found distraction from positive emotions was associated with lower well‐being only among Germans and not Japanese participants (Schunk, Trommsdorff, and König‐Teshnizi 2022). In contrast, distraction was not significantly associated with depression across cultural groups (Aldwin and Greenberger 1987). One study examined worry and found its use did not differ between East Asian and Western cultures (Nagulendran and Jobson 2020). For East Asian participants, worry did not significantly correlate with PTSD symptoms, but for their Western counterparts, worry was linked to greater PTSD severity (Nagulendran and Jobson 2020).

#### 2.7.1 Control Appraisals

##### 2.7.1.1 Cultural Differences in Use of Control Appraisals

The most commonly investigated appraisal related to sense of control (*k* = 12). Four studies found no significant cultural differences in primary control (Engelbrecht and Jobson 2014; Hamamura and Mearns 2019; Turner 2022; Xiu et al. 2016). Two studies reported higher primary control among East Asians compared to their Western counterparts (Chang and Yang 2024; Jobson et al. 2024), whereas three studies found lower perceived control among East Asian participants (Engelbrecht and Jobson 2014; O'Connor and Shimizu 2002; Peng 1995). Two studies found no cultural differences in fatalistic appraisals (Au et al. 2012; Xiu et al. 2016). However, several studies reported that East Asians more strongly endorsed negotiable fate (Au et al. 2012), secondary and fatalistic appraisals (Jobson et al. 2024; Peng 1995; Turner 2022), cultural beliefs emphasising the adaptive value of adversity (Jobson et al. 2024) and attributed blame as being a central component in identity (Turner 2022). Contrastingly, one study found that whereas American women valued secondary control more than personal control, Japanese women viewed both as equally important (Morling et al. 2003).

##### 2.7.1.2 Cultural Influences on the Associations Between Control Appraisals and Mental Health

Seven studies indicated that a perceived lack of control was associated with poorer mental health (lower self‐esteem, greater existential loneliness, depression and reduced well‐being) across cultures (Au et al. 2012; Chung et al. 2020; Hamamura and Mearns 2019; Jobson et al. 2024; Krause et al. 1991; Peng 1995; Turner 2022). In contrast, two studies found no association between perceived control (personal control and fate control) and mental health (distress and grief symptoms) across cultures, both cross‐sectionally and longitudinally (Morling et al. 2003; Xiu et al. 2016). Several studies (*k* = 6) suggested primary control was uniquely associated with better mental health (less PTSD and depressive symptoms, perceived stress, grief severity and higher life satisfaction) among Western but not East Asian individuals (Engelbrecht and Jobson 2014; Jobson et al. 2024; Krause et al. 1991; O'Connor and Shimizu 2002; Peng 1995; Xiu et al. 2016). Conversely, forms of secondary control were uniquely beneficial for East Asians (Au et al. 2012; Jobson et al. 2024).

A recent study by Jobson et al. (2024) highlighted the nuanced role of cultural values in shaping the impact of control appraisals on mental health. The negative association between Chinese cultural beliefs about adversity and PTSD symptoms was only significant among those with higher interdependence and holistic thinking styles, whereas the positive relationship between fatalism and PTSD severity was only significant for those with higher independence and holistic thinking tendency. Nonetheless, regardless of self‐construal and thinking style, for Chinese Australians, the lack of secondary control was associated with more PTSD symptoms, whereas for European Australians, the lack of primary control appraisals was associated with more PTSD symptoms.

##### 2.7.1.3 Control Appraisals Summary

There was some evidence that East Asians tended to endorse greater secondary control, fatalistic beliefs and adversity‐related cultural meanings, whereas Western samples showed greater use of personal control. Overall, lower perceived control was generally linked to poorer mental health across cultures, with primary control particularly important for Western individuals and secondary control important for East Asians.

#### 2.7.2 Other Appraisal Types

##### 2.7.2.1 Cultural Differences in Use of Other Appraisal Types

As outlined in Table S3, East Asians tended to endorse more traditional values (Aldwin and Greenberger 1987), greater social complexity, lower social cynicism (Xiu et al. 2016) and more pessimistic appraisals (Adams 2003) and were more likely to believe that change is inevitable and nonlinear (Ji and Wang 2024) than Western participants. Whereas Ji et al. (2022) found East Asians had a stronger tendency to engage in meaning‐making for stressful events, Turner (2022) found Japanese participants reported lower meaning‐making compared to their Western counterparts. Engelbrecht and Jobson (2014) found Asian participants perceived positive memories as more pleasant, trauma memories as more legitimate and reported less anticipated effort appraisals and motivation for attentional engagement with future events than their British counterparts. Regarding self‐related appraisals, several studies found East Asians reported the self as less favourable or consistent (Cai et al. 2007; Engelbrecht and Jobson 2014; Gage et al. 2015).

##### 2.7.2.2 Cultural Influences on the Associations Between Other Types of Appraisals and Mental Health

Culturally specific associations were observed for certain appraisal types: Perceiving parents as endorsing traditional values was associated with higher depression among Koreans than Western participants (Aldwin and Greenberger 1987), social cynicism was uniquely associated with greater prolonged grief among Chinese individuals than Western individuals (Xiu et al. 2016), greater attentional engagement with an event was negatively associated with PTSD symptoms among East Asian but not Western participants (Engelbrecht and Jobson 2014), appraising a stressful event as a loss was positively associated with anxiety among East Asians but not Western participants (Bjorck et al. 2001) and a stronger tendency to reflect on the value of stressful events was associated with greater positive affect across cultures, with this relationship being stronger among Chinese participants than Euro‐Canadians (Ji et al. 2022). Several associations were found between appraisals (negative events being central to identity, Zaragoza Scherman et al. 2015; positive future outlook, Chang and Yang 2024; positive self‐appraisals, Cai et al. 2007; Ji and Wang 2024; Nauta et al. 2010; endorsement of undesirable self traits, Gage et al. 2015) and mental health across both cultural groups, whereas two studies found no associations between appraisals (meaning making, Turner 2022; pessimism, Adams 2003) and mental health in both groups. Engelbrecht and Jobson (2014) found that whereas self‐directed accountability (e.g., negative self) was associated with higher PTSD symptoms across both cultural groups, accountability directed towards others or external constructs (e.g., negative world views) was maladaptive only among Western participants.

##### 2.7.2.3 Other Appraisal Types Summary

Compared to Western individuals, East Asians tended to endorse more traditional, dialectical and pessimistic beliefs and reported less favourable or consistent self‐views. Some appraisals showed culturally specific links with mental health (e.g., traditional values, social cynicism and attentional engagement), whereas others (e.g., identity centrality, future outlook and self‐appraisals) were associated with mental health across cultures.

## 3 Discussion

The aim of this study was to systematically examine cultural differences in the use and mental health benefits of emotion regulation among individuals from East Asian and Western cultural contexts.

### 3.1 Cultural Differences in the Use of Emotion Regulation Strategies

Based on the most robust and consistent empirical findings, this systematic review revealed five key findings relating to cultural differences in the use of emotion regulation strategies. First, East Asians tended to use avoidance and rumination more frequently than their Western counterparts. Furthermore, the use of these strategies was linked to greater levels of interdependence, endorsement of rejection avoidance and harmony seeking (Ogawa 2009; Schunk, Wong, et al. 2022). This aligns with theoretical accounts that posit East Asians as less motivated to downregulate negative emotions and upregulate positive emotions, as regulatory goals are driven by a desire to avoid failure to fulfil social obligations rather than the accomplishment of positive outcomes, such as happiness and pride (Mesquita and Albert 2007). Hence, East Asians may be more motivated to avoid negative emotions as opposed to approaching positive ones. Similarly, rumination may be used as a self‐distancing process to view negative emotions from its broader contextual and social environment and to facilitate insights for further adaptive development, rather than a fixated vessel for persistent negative thoughts and self‐doubt (Choi and Miyamoto 2023; de Vaus et al. 2018; Li et al. 2025).

Second, some studies demonstrated that East Asians engaged in greater use of suppression and less use of reappraisal than Western individuals. However, the findings were inconclusive, as other studies found no cultural group differences in use of these strategies. Although studies did not always reveal cultural group differences in use of these strategies, the degree to which individuals endorsed particular cultural values appeared to play a role in shaping suppression and reappraisal tendencies. For instance, those with higher levels of independent self‐construal tended to suppress less (Akutsu et al. 2016; Kalibatseva and Leong 2018; Tse 2017) and interpersonal‐related cultural values (i.e., harmony‐seeking and rejection avoidance) contributed to variations in suppression and reappraisal use even among individuals from Western cultural backgrounds (Schunk, Wong, et al. 2022). These findings reflect that for individuals who prioritise interdependence and place importance on social relationships and interpersonal harmony, emotional constraints may be exercised to reduce interpersonal burden and reappraisals may be engaged to modify emotions in order to adapt to the social environment (Ford and Mauss 2015; Jobson et al. 2025). This evidence highlights the need to consider one's endorsement of particular cultural values, rather than merely relying on cultural groupings.

Third, findings regarding acceptance were mixed. Contrary to theories that suggest East Asian individuals are more accepting of emotions (de Vaus et al. 2018), results regarding cultural differences in the use of acceptance were inconsistent. Instead of concluding the absence of cultural influences, it is perhaps fairer to consider acceptance as an unconsciously learnt response, embedded deeply in traditional Taoist and Buddhist philosophies that emphasise the surrendering to emotions for inner peace and the power of refraining from active regulation (Lin et al. 2021). Among East Asians, acceptance may not always be a deliberate or consciously chosen strategy; rather, its use may be passive or automatic, hence more difficult to identify. Further, the measure of acceptance in the included studies was largely heterogeneous, reflecting variations in how acceptance was captured across samples. Notably, qualitative study revealed that acceptance holds unique cultural meanings within Japanese people's identity, in the context of navigating challenging life events (Turner 2022). This highlights the need for further research examining how acceptance is conceptualised and operationalised in East Asian cultures, as well as considering the potential mediating roles of traditional philosophical values (e.g., endorsement of Taoist values).

Fourth, for certain emotion regulation strategies—interpersonal emotion regulation, problem‐solving, distraction and worry—the evidence was limited, dated and inconclusive. Given the emphasis on interconnectedness, it is crucial for future research to examine the use of interpersonal emotion regulation among East Asians, specifically, whether interpersonal relationships, central to the collectivistic cultures (Mesquita and Albert 2007), function as adaptive or maladaptive forms of regulation. Similarly, East Asians may be more reluctant to engage in problem‐focused strategies due to the potential interpersonal burden it may impose and the risk of disrupting social relationships. Further investigation of these culturally shaped regulatory preferences is essential in advancing a more culturally nuanced understanding of emotion regulation.

Fifth, regarding appraisals, East Asians were found to endorse more secondary control appraisals that reinforce adapting the self to the environment. They also had a tendency to attribute negative (as opposed to positive) experiences to the self. These tendencies reflect the Taoist and Buddhist views regarding suffering and adverse events as a natural part of life, holding motivation for positive transformations and growth (Lin et al. 2021). Further, East Asians endorsed more traditional values and social complexity; they tended to view themselves less favourably, exhibit a more pessimistic outlook and show greater acceptance and expectation of change. These patterns may be understood in light of cultural attitudes towards negative emotions—where traditional philosophical values promote the importance of negative emotions and hence consider them not as threatening but rather as constructive insights for growth (de Vaus et al. 2018; Lin et al. 2021). Together, these orientations towards adversity and negative self or worldviews reflect a form of cultural resilience, wherein negativities are embraced as equally valuable aspects of life. Rather than being suppressed or avoided, they are regarded as central to the self, endured and utilised as instruments for self‐improvement.

### 3.2 Cultural Influences on the Effectiveness of Emotion Regulation Strategies

A large body of evidence consistently indicated that certain emotion regulation strategies—suppression, avoidance and rumination—were associated with poorer mental health. However, the strength of these associations tended to be less pronounced and at times absent or even beneficial, among East Asians. There was also some evidence, although limited, suggesting the adverse effects of distraction and worry on mental health were observed in individuals from Western cultural contexts but not among East Asians (Nagulendran and Jobson 2020; Schunk, Trommsdorff, and König‐Teshnizi 2022). These findings indicate that the negative impacts of these putatively maladaptive strategies may be mitigated by their cultural congruence (or incongruence) with values, such as emotional constraints and social harmony (Markus and Kitayama 1991; Mesquita and Albert 2007). For instance, suppressing emotions may reduce the burden on interpersonal relationships and align with culturally valued regulatory goals (Jobson et al. 2025). Similarly, avoidance may help preserve relational harmony by preventing interpersonal conflict, whereas rumination may provide self‐evaluative benefits that promote personal growth through private processing of negative emotions, thereby minimising social disruption (Choi and Miyamoto 2023; Mesquita and Albert 2007). Therefore, the use of these strategies may foster a sense of relational appropriateness and reduce interpersonal stress. However, definitive conclusions should not be drawn at this stage, as the detrimental long‐term outcome needs to outweigh its short‐term benefits for a given strategy to be considered adaptive (Werner and Gross 2010), and the current literature lacks sufficient longitudinal research to fully understand the long‐term outcomes associated with these strategies among East Asians.

Within the construct of suppression, the effectiveness of the strategy appears to vary depending on the emotional valence, the type and quality of suppression. For instance, the inability to suppress emotions (i.e., uncontrolled expression of emotions) appeared as more harmful for East Asians (Schunk, Trommsdorff, and König‐Teshnizi 2022). Similarly, although higher frequency of suppression was linked to negative outcomes for American participants, higher perceived ability to suppress emotions in response to situational needs was found beneficial across cultures (Chen et al. 2020), suggesting the need for further studies to consider daily situational contexts when examining the effectiveness of a given strategy.

Although most research on interpersonal emotion regulation found no benefits on mental health, the majority of this research was dated (e.g., Aldwin and Greenberger 1987). Emerging research, using quasiexperimental designs, suggests potential adaptiveness of interpersonal emotion regulation on short‐term psychophysiological outcomes for East Asian participants (Liddell and Williams 2019), warranting further investigations. Surprisingly, limited cross‐sectional and longitudinal evidence suggested a potential maladaptive role of acceptance on the mental health among East Asian individuals, but acceptance appeared an adaptive strategy for their Western counterparts. This finding is counter to cultural predictions based on East Asian philosophies of Taoism and Buddhism, which posit that acceptance involves a harmonious engagement with emotions and the alleviation of suffering through nonresistance and embracement, which offer a source of coping in navigating stress by fostering psychological balance and inner peace (Lin et al. 2021). However, one factor that might explain these contrasting findings is that acceptance may be informed by fatalistic appraisals, a belief that life is predetermined by forces beyond one's control, and emphasises the acceptance of circumstances as inevitable (Maercker et al. 2019). Fatalism was found to be associated with negative mental health outcomes (Au et al. 2012; Jobson et al. 2024), as it may foster the perceptions of inability to manage stressful events, potentially contributing to maladaptive consequences (Jobson et al. 2024).

In regard to appraisals, perceived lack of control tended to be associated with poorer mental health across cultures. Distinctions highlighted that the perceived inability to change the circumstances to fit one's personal goals may be more detrimental for Western individuals, whereas the inability to change the self and adapt to environmental needs may have more negative impacts on the mental health of East Asians. Importantly, emerging research revealed that control appraisals may hold different significance within specific cultural contexts; the extent to which individuals ascribe to certain cultural values (e.g., self‐construal and holistic thinking style) is an important consideration when evaluating the adaptive functioning of some appraisals (i.e., fatalism and Chinese cultural beliefs about adversity) (Jobson et al. 2024). Additionally, self‐directed accountability and less favourable self‐appraisals, which were found to be more pronounced among East Asian individuals, tended to be linked to more maladaptive mental health, suggesting attribution biases (particularly those attributing negativity to the self) may be an important clinical target when working with individuals from East Asian cultural backgrounds (Choi and Miyamoto 2023).

### 3.3 Theoretical Implications

The findings of this review highlight the importance of integrating cultural frameworks into both models of emotion regulation and models of mental health emphasising emotion regulation. The observed differences between members of East Asian and Western cultures suggest that prevailing Western‐centric theories of emotion regulation (Gross 2015; Koole 2009) may not fully capture the nuanced ways in which cultural norms, values and beliefs shape emotional processes. Moreover, transdiagnostically, models of mental health disorders emphasise the centrality of maladaptive emotion regulation in both aetiology and maintenance of symptomatology (e.g., Cisler et al. 2010; Ehlers and Clark 2000; Ehring 2021). Yet, similar to theories of emotion regulation, these models remain Western‐centric and rarely consider the influence of culture on the expression and regulation of emotion. Therefore, incorporating explicit cultural frameworks, such as theory of self‐construal (Markus and Kitayama 2010), the three teachings of East Asia (i.e., Confucianism, Taoism and Buddhism; Lin et al. 2021), thinking styles (e.g., holistic vs. analytic; Jobson et al. 2024), relational principles (e.g., harmony seeking and rejection avoidance; Schunk, Wong, et al. 2022), is essential to provide valuable insights into the regulatory goals that underlie the effectiveness of emotion regulation strategies, particularly in relation to the development and maintenance of mental health disorders. This, in turn, helps clarify why certain emotion regulation approaches are more or less adaptive in particular cultural contexts. The emerging literature also highlights the need for theoretical models to move beyond simplistic group‐based comparisons and incorporate cultural values as dynamic, individual‐level constructs, which can more accurately account for variability in emotion regulation outcomes across populations and situational demands.

### 3.4 Clinical Implications

Current evidence reinforces the importance of integrating cultural considerations into clinical practices targeting emotion regulation, in particular, when assessing the adaptive/maladaptive functioning of emotion regulation, developing formulations and when targeting emotion regulation in treatment. Supported by a strong body of evidence, East Asian samples appear to use a range of emotion regulation strategies that vary from their Western counterparts. When regulatory processes do not align with the given sociocultural context, it may lead to negative mental health consequences (Ford and Mauss 2015). Nonetheless, as suggested by emerging findings (e.g., Jobson et al. 2024; Schunk, Wong, et al. 2022), individual level cultural values (rather than merely cultural background or heritage) may influence distinct regulatory processes that differentially impact mental health (Hirano and Ishii 2024). Hence, rather than assuming homogeneity within a specific cultural group, it is more valuable to consider the extent to which an individual ascribes to certain cultural values, providing meaningful insights to an individual's regulatory goals (Ford and Mauss 2015; Mesquita and Albert 2007). Evidence on the role of these values (e.g., self‐construal) in driving cultural differences in emotion regulation and their effectiveness is compelling, highlighting the need for clinical practice and future research to unpack key cultural mediating factors, such as core values, belief systems (e.g., Taoism, Buddhism and Confucianism; Lai et al. 2025) and culturally shared significant events (e.g., collective trauma and shared developmental environments), that shape emotional processes and have the potential to inform early interventions targeting emotion regulation difficulties.

Evidence‐based psychological interventions (e.g., Cognitive Behavioural Therapy [CBT], Acceptance and Commitment Therapy, trauma‐focused interventions and Dialectical Behavioural Therapy) target emotion regulation. However, the foundation of these models, particularly the gold standard of CBT, is grounded in Western cultural values of independence, regaining primary control and assertiveness; such processes may not be congruent with East Asian cultural context that prioritise interdependence, secondary control and indirect communication styles (Huey et al. 2023). Systematic and meta‐analytic evidence suggest that, with appropriate cultural adaptation/modification, mainstream psychological interventions can derive benefits for individuals from non‐Western cultural backgrounds (Huey et al. 2023; Li et al. 2023). In line with the findings of this review and the three broad models proposed by Huey et al. (2023), incorporating culture in clinical practice may look like (1) cultural competency training for clinicians (e.g., orienting clinicians to cultural beliefs, such as secondary control appraisal), (2) training clinicians in cultural adaptation strategies and (3) adopting a person‐centred approach that meets a client at their individual cultural context (e.g., considering individual endorsement of interdependence).

### 3.5 Methodological Limitations

Consistent limitations in the literature were observed, which are essential to consider for advancing future cross‐cultural emotion regulation research. First, cultural inclusion and exclusion criteria were not well operationalised across multiple studies. This resulted in heterogeneity of Western comparison groups, which often included individuals from a variety of cultural backgrounds, which may have confounded the cross‐cultural findings. Compounding this, the inclusion of East Asian participants across the studies also displayed considerable heterogeneity, with culture being based on ethnicity, country of residence, nationality, race, ancestry, mother tongue and birthplace. This is potentially problematic, as individuals from diverse East Asian regions may endorse cultural values in markedly different ways which impact emotion regulation.

Second, caution needs to be exercised when considering findings relating to acceptance, problem‐solving and interpersonal emotion regulation, as the limited evidence‐base is relatively dated and may not reflect contemporary theories and methodological approaches to investigating emotion regulation. Third, the current literature largely centres around Western‐driven frameworks of emotion regulation (e.g., suppression *k* = 18; reappraisal *k* = 15; avoidance *k* = 13; and control appraisal *k* = 16) and often focuses on strategies where East Asians are presumed to differ, rather than strategies that are informed by East Asian cultural values (e., acceptance informed by Taoist and Buddhist values, interpersonal emotion regulation informed by Confucianism). Compounding this, measures used across studies for not only emotion regulation but also mental health outcomes were defined, developed and normed based on Western mental health frameworks. Only 40% of the included studies conducted psychometric assessments to test measurement invariance across cultural groups, representing a critical concern given the direct cross‐cultural comparison focus of the included articles. Without establishing measurement equivalence, it remains difficult to conclude whether observed cultural differences reflect true variation in emotion regulation constructs or artefacts of nonequivalent measurement. Although measurement invariance studies have reported construct invariance across Asian and Western samples for commonly used emotion regulation scales such as the Emotion Regulation Questionnaire (ERQ; Gross and John 2003; e., Chan et al. 2024), measurement invariance, even if established, may mask genuine cultural differences in emotion regulation processes.

### 3.6 Future Directions

Future research would benefit from extending beyond predominantly nonclinical community and student‐focused samples to include clinical populations. This would enable findings to inform culturally responsive insights that can be directly applied to assessment, formulation and intervention in clinical practice. Second, greater operational specificity in defining ‘culture’ is warranted. Rather than relying on broad geographic or ethnic/racial group comparisons, future studies should clearly articulate how culture is operationalised. Third, increased methodological rigour is needed, including more consistent use of validated cross‐culturally equivalent measures, and formal testing of measurement invariance. Fourth, future research would benefit from longitudinal methodologies to clarify whether the short‐term benefits and attenuated maladaptive effects of certain strategies (e.g., suppression, avoidance and rumination) are sustained over time.

Fifth, future research should consider including measures of individual‐level endorsement of cultural values and move beyond categorical cultural group comparisons to provide a more nuanced understanding of how culture shapes emotion regulation processes and their implications for mental health. Sixth, findings revealed nuances within specific regulatory processes, such as regulation frequency and ability (Chen et al. 2020), which give rise to the variability in its effectiveness. These nuances highlight the importance of considering the specific regulatory context, thereby warranting future research into the contextual and momentary use and effectiveness of emotion regulation strategies across cultures (Chen et al. 2020; Qiu et al. 2025). Finally, future research may consider exploring within‐strategy nuances, such as regulating positive versus negative emotions, as they appeared to carry important cultural implications when evaluating the effectiveness of specific regulatory processes.

### 3.7 Study Limitations

The current review has several limitations worth noting. First, the conducted literature search was limited to English‐language databases and did not identify eligible non‐English publications. This introduces potential language bias, as relevant studies published in other languages may have been excluded. Although this decision was made due to challenges in conducting consistent quality assessments across differing reporting standards, it may have limited the comprehensiveness and cultural representativeness of the findings. Second, many of the studies included in the review focused on student samples, which limits generalisability for clinical samples. Third, a limitation of this review is cultural essentialism, whereby the review has overstated group differences and portrayed cultures as homogeneous, thereby overlooking within‐culture variability and contextual influences. Thus, future studies should consider cultural variables at the individual level, within‐culture variability, intersectional identities and contextual factors and employ designs that conceptualise culture and emotion regulation as dynamic. Fourth, there was heterogeneity in some of the emotion regulation constructs, such as suppression, problem‐solving and interpersonal emotion regulation, which may have undermined the nuances of certain constructs. Lastly, whereas we included theses and dissertations (*k* = 5), other forms of grey literature (e.g., conference proceedings and unpublished reports) were not included. Hence, interpretations should be made with consideration of publication bias.

## 4 Conclusion

This systematic review shed light on the cultural differences in emotion regulation use and the associated mental health benefits between individuals from East Asian and Western cultural contexts. Results challenge the assumed universality of emotional processes and their adaptive/maladaptive functioning and suggest the congruence (or incongruence) of a given emotion regulation strategy with one's cultural context is an important consideration in influencing its effectiveness. This review provides insights for future research and clinical practice to consider the role of sociocultural (e.g., cultural groupings and social relationships) and individual level (e.g., level of self‐construal) factors that shape these differences.

## Funding

This study was funded by the National Health and Medical Research Council (Ideas Grant APP2010654).

## Ethics Statement

The authors have nothing to report.

## Conflicts of Interest

The authors declare no conflicts of interest.

## Supporting information


**Table S1:** Complete list of search terms.
**Table S2:** Quality assessment items and mean and standard deviations.
**Table S3:** Detailed findings on cultural differences in emotion regulation use.
**Table S4:** Detailed findings on cultural differences in the associations between emotion regulation and mental health.

## Data Availability

No new data are generated. Relevant review data are available on request from the corresponding author.

## Endnotes

^1^
The East Asian cultural group could be individuals residing within an East Asian country or within another country (e.g., as a migrant or international student).
