# Supplementary material for: Exploring Cultural Influences in the Associations Between Emotion Regulation and Mental Health: A Systematic Review Comparing East Asian and Western Cultural Contexts
Source: Clin Psychol Psychother. 2026 Apr 21;33:e70276. doi: 10.1002/cpp.70276 (PMC13097086; doi:10.1002/cpp.70276)
Supplement: Supplementary file 1 — Table S1: Complete list of search terms. Table S2: Quality assessment items and mean and standard deviations. Table S3: Detailed findings on cultural differences in emotion regulation use. Table S4: Detailed findings on cultural differences in the associations between emotion regulation and mental health. [file CPP-33-e70276-s001.docx]

**Exploring Cultural Influences in the Associations between Emotion Regulation and Mental Health: A Systematic Review Comparing East Asian and Western Cultural Contexts**

Supplementary Material

*Table 1.* Complete list of search terms

| **Database** | **Search Terms** |
| --- | --- |
| **PsychINFO (Ovid)** | Cross cultural differences/  Cross cultural psychology/  Cultural diversity/  Ethnic diversity/  "Racial and ethnic differences"/  ((race or racial or ethnic* or cultur*) adj3 (psychology or difference* or diversity or comparing or comparison)).mp.  Chinese cultural groups/  Japanese cultural groups/  Korean cultural groups/  (East Asia* or Chinese or China or Japan* or Korea* or taiwan* or Hong Kong or macau or mongol*).mp.  Cognitive appraisal/  psychological adaptation  Cognitive processes/  Ethnic values/  (cognitive* adj1 (appraisal or perceiv* or perception* or process* or restructur*)).mp.  belief*.mp  (ethnic or cultur*) adj1 value*.mp.  Emotional regulation/  Coping behaviour/  Coping style/  Emotional control/  (emotion* or affect*) adj2 (dysregulat* or regulat* or difficult* or control*)  Cope  Coping  Experiential avoidance/  Avoidance/  Thought suppression/  avoidance adj2 (behav* or emotion* or experiential)  (suppress* or conceal* or hid* or control*) adj2 (thought or cognitive or emotion* or expressive or affect*)  Rumination (cognitive process)/  problem solving/  distraction/  reapprais*  positive* refram*  acceptance  ruminat*  worr*  problem solv*  distraction |
| **MEDLINE ALL (Ovid)** | Cross-cultural comparison/  Cultural Characteristics/  Cultural diversity/  Racial Groups/  ((race or racial or ethnic* or cultur*) adj3 (psychology or difference* or diversity or comparing or comparison)).mp.  East Asian people/  Asia, Eastern/  China/  Japan/  Korea/  Hong Kong/  Taiwan/  Macau/  (East Asia* or Chinese or China or Japan* or Korea* or taiwan* or Hong Kong or macau or mongol*).mp.  Adaptation, psychological/  psychological adaptation  (cognitive* adj1 (appraisal or perceiv* or perception* or process* or restructur*)).mp.  belief*.mp  (ethnic or cultur*) adj1 value*).mp.  Emotional regulation/  Coping skills/  (emotion* or affect*) adj2 (dysregulat* or regulat* or difficult*)  Cope  Coping  avoidance adj2 (behav* or emotion* or experiential)  (suppress* or conceal* or hid* or control*) adj2 (thought or cognitive or emotion* or expressive or affect*)  Rumination, cognitive/  Problem Solving/  reapprais*  positive* refram*  acceptance  ruminat*  worr*  problem solv*  distraction |
| **Scopus** | (race or racial or ethnic * or cultur*) w/3 (psychology or difference* or diversity or comparing or comparison)  "East* Asia*"  China or Chinese or Japan* or Korea* or "Hong Kong" or Taiwan* or Macau or Mongol*  "psychological adaptation"  (cognitive*) w/3 (apprais* or perceiv* or perception* or process* or restructur*)  belief*  (ethnic or cultur*) w/2 value*  (emotion* or affect*) w/3 (regulat* or dysregulat* or difficult*)  cope  coping  avoidance w/2 (behav* or emotion* or experiential)  (suppress* or conceal* or hid* or control*) w/2 (thought or cognitive or emotion* or expresive or affect*)  reapprais*  "positive* refram*"  acceptance  ruminat*  worr*  "problem solv*"  distraction |
| **PTSDpubs** | MAINSUBJECT.EXACT("Ethnic identity")  (race or racial or ethnic * or cultur*) n/3 (psychology or difference* or diversity or comparing or comparison)  MAINSUBJECT.EXACT("Chinese")  MAINSUBJECT.EXACT("Japanese")  MAINSUBJECT.EXACT("Koreans")  MAINSUBJECT.EXACT("Mongolians")  MAINSUBJECT.EXACT("Tibetans")  "East Asia*" or Chinese or China or Japan* or Korea* or taiwan* or "Hong Kong" or macau or mongol*  MAINSUBJECT.EXACT("Adaptability")  "psychological adaptation"  MAINSUBJECT.EXACT("Cognitive restructuring")  MAINSUBJECT.EXACT("Culture-Bound Syndromes")  MAINSUBJECT.EXACT("Cognitive processess")  (cognitive*) n/3 (apprais* or perceiv* or perception* or process* or restructur*)  belief*  MAINSUBJECT.EXACT("Cultural values")  (ethnic or cultur*) n/2 value*  MAINSUBJECT.EXACT("Emotion regulation")  MAINSUBJECT.EXACT("Coping Behavior")  (emotion* or affect*) n/3 (regulat* or dysregulat* or difficult*)  noft(cope)  noft(coping)  MAINSUBJECT.EXACT("Suppression")  MAINSUBJECT.EXACT("Avoidance")  avoidance n/2 (behav* or emotion* or experiential)  (suppress* or conceal* or hid* or control*) n/2 (thought or cognitive or emotion* or expresive or affect*)  MAINSUBJECT.EXACT("Rumination")  reapprais*  "positive* refram*"  acceptance  ruminat*  worr*  "problem solv*"  distraction |
| **Social Science Database** | MAINSUBJECT.EXACT("Cross-culture psychology")  MAINSUBJECT.EXACT("Cross cultural studies")  MAINSUBJECT.EXACT("Cultural differences")  MAINSUBJECT.EXACT("cultural studies")  MAINSUBJECT.EXACT("Ethnic studies")  (race or racial or ethnic * or cultur*) n/3 (psychology or difference* or diversity or comparing or comparison)  MAINSUBJECT.EXACT("Chinese culture")  MAINSUBJECT.EXACT("Japanese culture")  MAINSUBJECT.EXACT("Korean culture")  "East Asia*" or Chinese or China or Japan* or Korea* or taiwan* or "Hong Kong" or macau or mongol*  MAINSUBJECT.EXACT("Adaptation")  MAINSUBJECT.EXACT("Adjustment")  "psychological adaptation"  (cognitive*) n/3 (apprais* or perceiv* or perception* or process* or restructur*)  belief*  MAINSUBJECT.EXACT("cultural values")  (ethnic or cultur*) n/2 value*  MAINSUBJECT.EXACT("Emotional regulation")  MAINSUBJECT.EXACT("Coping")  (emotion* or affect*) n/3 (regulat* or dysregulat* or difficult*)  cope  coping  MAINSUBJECT.EXACT("Avoidance behavour")  avoidance n/2 (behav* or emotion* or experiential)  (suppress* or conceal* or hid* or control*) n/2 (thought or cognitive or emotion* or expressive or affect*)  MAINSUBJECT.EXACT("Problem Solving")  reapprais*  "positive* refram*"  MAINSUBJECT.EXACT("Acceptance")  acceptance  ruminat*  MAINSUBJECT.EXACT("Worry")  worr*  "problem solv*"  distraction |

*Table 2.* Quality assessment items and mean and standard deviations

|  | **Item** | ***Percentage of study which met criteria*** | ***SD*** |
| --- | --- | --- | --- |
| 1 | Was ethical approval or consent of participants obtained? | 64% | 0.48 |
| 2 | Was the research question or objective in this paper clearly stated? | 100% | 0 |
| 3 | Was the study design appropriate for the stated aim(s)? | 98% | 0.14 |
| 4 | Was the study population clearly specified and defined? | 83% | 0.38 |
| 5 | Were inclusion and exclusion criteria for being in the study clearly defined? | 36% | 0.48 |
| 6 | Was a sample size justification, power description, or variance and effect estimates provided? | 25% | 0.43 |
| 7 | Were the study procedures adapted to enhance cultural responsiveness? | 62% | 0.49 |
| 8 | Were the emotion regulation measures clearly defined, valid, reliable, and implemented consistently across all study participants? | 92% | 0.27 |
| 9 | Were the mental health measures clearly defined, valid, reliable, and implemented consistently across all study participants? | 96% | 0.19 |
| 10 | Was an explanation for missing data given? | 25% | 0.43 |
| 11 | Were key potential confounding variables measured and adjusted statistically for their impact? | 83% | 0.38 |
| 12 | Did the study assess measurement invariance to ensure constructs were measured equivalently across groups? | 40% | 0.49 |
| 13 | Was appropriate statistical analysis used? | 92% | 0.27 |
| 14 | Were the authors’ discussions and conclusions justified by the results? | 98% | 0.14 |
| 15 | Were the limitations of the study discussed? | 98% | 0.14 |

*Note.* Close to half of the above criteria was not applicable to one qualitative study (i.e., Chung et al., 2020), hence was not included in this analysis.

*Table 3.* Detailed findings on cultural differences in emotion regulation use

| **Study** | **Study Design and Sample Characteristics** | **Emotion Regulation Construct/s** | **Key Findings (Cultural Differences in Emotion Regulation)** |
| --- | --- | --- | --- |
| **Adams (2003)** | Cross Sectional  Spousal Caregivers of Partner with Dementia;  41 Japanese Americans  67 Anglo-Americans | Escape/Avoidance Coping  Seeking Social Support  Active/Planful Coping  Positive Appraisal  Self-Efficacy Appraisal  Spiritual Support  Perfectionistic Appraisal  Pessimistic Appraisal  Lack-of-Support Appraisal | Japanese-American caregivers scored significantly higher on pessimistic appraisal and seeking social support than Anglo-American.  No significant differences found in other appraisal or coping domains. |
| **Akutsu et al. (2016)** | Cross Sectional  General Community;  1027 Japanese  1255 Americans | Anger Regulation  *(Anger In, Anger Out, Anger Control)* | For both Japanese and Americans,   - Independent self-construal was positively related to anger-out and not related to anger control. - Interdependent self-construal was not related to anger-in, negatively related to anger-out.   Independent self-construal was negatively related to anger-in for Americans, but not related for Japanese.  Interdependent self-construal was positively related to anger control for Americans but not related for Japanese. |
| **Aldwin & Greenberger (1987)** | Cross Sectional  University Sample;  61 Koreans  69 Caucasian Americans | Distraction  Reappraisal  Problem- Solving  Emotional Expression  Acceptance  Interpersonal Emotion Regulation  Seek Spiritual Comfort  Appraisal of Cultural Values | Koreans were more likely to express emotions than Caucasians.  Koreans endorsed traditional values more highly and perceived their parents’ values as more traditional than Caucasians.  No significant group difference in endorsement of modern values, perception of parents' stance concerning modern values, and other emotion regulation strategies. |
| **Au et al., (2012)** | Cross Sectional  University Sample;  168 Chinese  165 Americans | Negotiable F ate  Fatalism | Chinese believed more strongly in negotiable fate than Americans, but did not differ in fatalism. |
| **Bjorck et al., (2001)** | Cross Sectional  College students/Young adults in Church community;  93 Korean Americans  86 Caucasian Americans | Confrontive Coping  Distancing  Self-Controlling  Seeking Social Support  Escape-Avoidance  Problem Solving  Positive Reappraisal  Accepting Responsibility  Appraisal *(appraising a stressful event as threat/challenge/loss)* | Koreans reported more passive coping behaviours (acceptance responsibility, religious coping, distancing, and escape-avoidance) and appraised events as greater challenges than Caucasians.  No significant group difference in other strategies. |
| **Bonanno et al. (2005)** | Longitudinal Study  General Community;  71 Chinese  68 Americans | Deliberate Grief Avoidance | Chinese reported significantly greater deliberate grief avoidance than Americans. |
| **Cai et al. (2007)** | Cross Sectional  University Sample;  35 Chinese  36 Americans | Cognitive Self-Evaluations | Chinese participants appraised themselves (self-evaluations) less favourably than Americans. |
| **Chang & Yang (2024)** | Cross Sectional  University Sample;  357 Chinese  320 Americans | Positive Problem Orientation (*perception that problems encountered in life are solvable*)  Negative Problem Orientation (*perception that problems encountered in life are difficult to solve*)  Rational Problem S olving  Avoidance | Chinese reported significantly higher positive problem orientation, negative problem orientation, rational problem solving and avoidance. |
| **Chataway & Berry (1989)** | Cross Sectional  University Sample;  42 HKC  43 French-Canadians  42 English-Canadians | Problem Solving  Wishful Thinking  Detachment  Seeking Social Support  Positive Thinking  Self- Blame  Tension- Reduction  Withdrawal | English were significantly more inclined to use tension reduction techniques (e.g., eating, taking drugs, or exercising) than the Chinese or French.  The French engaged in more positive thinking than the Chinese or English.  No significant group difference in other strategies. |
| **Chen et al. (2020)** | Cross Sectional  General Community;  192 Chinese  200 Americans | Suppression Frequency  Suppression Ability | Chinese and American did not differ significantly in suppression frequency and ability. |
| **Choi & Miyamoto (2023)** | Cross Sectional  University Sample;  123 East Asian  142 European Americans | Rumination | East Asians reported ruminating more than European American. |
| **Chung et al. (2020)** | Qualitative  Geriatric Sample Living in Long-term Care Facilities;  13 HKC  9 Swedes | Appraisal of Control  Reframing  Acceptance  Reflection | No comparison reported. |
| **Corey & Allen (2013)** | Cross Sectional  University Sample;  96 Taiwanese Chinese  73 Chinese Americans 65 Caucasian Americans | Active Coping | Caucasian Americans reported significantly greater use of active coping strategies than Taiwanese.  Independent self-construal was correlated to more active coping.  Interdependent self-construal was not correlated with active coping. |
| **Engelbrecht & Jobson (2014)** | Cross Sectional  University Sample;  41 Chinese/East Asian  34 British | Cognitive Appraisals  *(pleasantness, legitimacy, anticipated effort, norm-self compatibility, attentional activity)*  Trauma- Specific Appraisals *(negative self, negative world, self blame)* | East Asians appraised memories as being more pleasant, and perceived trauma memory as significantly more legitimate than the British.  The British reported significantly greater anticipated effort appraisals, norm-self compatibility appraisals, attentional activity appraisals, and felt more personally responsible for the pleasant memory than the East Asians.  No cultural difference on self-blame, negative self and negative world appraisals, perceived control, certainty appraisal, goal/need conduciveness, personal responsibility for the trauma memory, legitimacy of the pleasant memory, and coping ability. |
| **Gage et al. (2015)** | Cross Sectional  General Community;  122 East Asians  172 British | Desirable T ypes of S elf-C onsistency (*consistently endorsing desirable characteristics and consistently denying undesirable characteristics*)  Undesirable T ypes of S elf-C onsistency (*consistently endorsing undesirable characteristics*) | The ​​British reported higher overall self-consistency, and consistently endorsing desirable characteristics than East Asians.  East Asians reported consistently endorsing undesirable characteristics more often.  No group differences regarding denying desirable and undesirable characteristics. |
| **Haliczer et al. (2020)** | Cross Sectional  Sample 1 (University)  54 East Asians  98 White  Sample 2 (General Community)  15 East Asians  63 White | Difficulties in E motion R egulation | Sample 1: The East Asian and White groups did not differ in ER difficulties.  Sample 2: East Asians reported less emotional nonacceptance than the White group. |
| **Hamamura & Mearns (2019)** | Cross Sectional  University Sample;  155 Japanese  176 Americans | Negative M ood R egulation E xpectancies (NMRE; *beliefs about one’s ability to improve one’s negative moods*)  Avoidant C oping | No significant group differences in NMRE and avoidant coping. |
| **He et al. (2021)** | Quasi- Experimental  University Sample;  40 East Asian Chinese  40 ‘White’ | Cognitive Reappraisal  Suppression (Pre-E xperiment Baseline) | No significant group differences in suppression or reappraisal. |
| **Hirano & Ishii (2024)** | Cross Sectional  General Community;  509 Japanese  503 Americans | Cognitive Reappraisal  Expressive Suppression  Avoidant Coping | Japanese reported higher suppression and avoidant coping but lower reappraisal compared to Americans. |
| **Ji & Wang (2024)** | Cross Sectional  University Sample;  222 Chinese  210 Euro-Canadians | Self-C ontinuity (*perception of oneself as a coherent and unified entity over time*)  Lay T heories of C hange (*lay beliefs about how events develop over time*) | Chinese reported higher self-continuity and lay theory of change than Euro-Canadians. |
| **Ji et al. (2022)** | Cross Sectional  University Sample;  403 Chinese  331 Euro-Canadians | Meaning in S tressful E xperience (MISE; *people’s beliefs in, and tendency to actively reflect on, the meaning and value of stressful experiences*) | Chinese scored higher than Euro-Canadians on the average MISE tendency, as well as on the two subscales (belief and reflection). |
| **Jobson et al. (2024)** | Cross Sectional  General Community;  129 Chinese Australians  140 European Australians | Primary Control  Secondary Control  Fatalistic Appraisal  Chinese Cultural Beliefs about Adversity | Chinese Australians endorsed greater primary and secondary control appraisal, Chinese cultural beliefs about adversity, and fatalism than European Australians. |
| **Kahn et al. (2017)** | Cross Sectional  University Sample;  256 Taiwanese  209 European Americans | Distress D isclosure (*one’s tendency to disclose versus conceal one’s distress across time and situations*) | No significant group differences in distress disclosure. |
| **Kalibatseva & Leong (2018)** | Cross Sectional  University Sample;  204 Chinese Americans  315 European Americans | Expressive Suppression  Cognitive Reappraisal | No significant cultural group differences in reappraisal and suppression.  For both groups:   - Independent and interdependent self-construal positively associated with cognitive reappraisal. - Interdependent self-construal was not related to expressive suppression.   Independent self-construal negatively associated with expressive suppression for Chinese Americans only, not correlated for European Americans. |
| **Krause et al. (1991)** | Cross Sectional  Geriatric Sample;  1517 Japanese  1523 Americans | Personal control | No comparison reported. |
| **Kwon & Kim (2019)** | Quasi-Experimental  University Sample;  213 HKC  196 Americans | Suppression | No comparison reported. |
| **Kwon et al. (2013)** | Cross Sectional  University Sample;  380 South Korean  384 Americans | Expressive Suppression  Cognitive Reappraisal  Rumination *(brooding, reflection)*  Anger Suppression | No significant group differences in expressive suppression and reappraisal.  Korean reported more reflective pondering and brooding but less anger suppression than Americans. |
| **Leung et al. (2011)** | Cross Sectional  University Sample;  250 HKC  144 Anglo-Australians | Avoidance Coping | HKC reported higher avoidance than Anglo-Australians. |
| **Liddell & Williams (2019)** | Quasi-Experimental  University Sample;  48 East Asian Australian  38 Western European Australian | Interpersonal Emotion Regulation *(enhancing positive affect, perspective taking, soothing, social modelling)*  Cognitive Reappraisal (Pre-experiment Baseline)  Expressive Suppression (Pre-experiment Baseline) | East Asians reported using interpersonal strategies more during experiment than Western Europeans, regardless of manipulation group.  No significant cultural group differences in reappraisal or suppression.  East Asians had higher perspective taking and social modelling and lower enhancing positive affect than Western Europeans. |
| **Morling et al. (2003)** | Longitudinal  Pregnant Women;  94 Japanese  56 Americans | Personal Influence (PI; *sense of personal influence, or control, over aspects of illness or stress*); Acceptance (AC)  Social Assurance Coping | American women ranked acceptance higher than personal influence and social assurance as a way to cope with common concerns of normal pregnancy (Acceptance > personal influence > social assurance).  Japanese women ranked social assurance higher than either acceptance or personal influence (social assurance > acceptance = personal influence). |
| **Nagulendran & Jobson (2020)** | Cross Sectional  General Community;  38 East Asian Australians  31 Caucasian Australians | Expressive Suppression  Cognitive Reappraisal  Habitual Thought Suppression  Habitual Worry  Habitual Experiential Avoidance  Habitual Rumination  Trauma-related Rumination  General Emotion Dysregulation | No comparison reported. |
| **Nagulendran et al. (2020)** | Quasi-Experimental  University Sample;  41 East Asian Australians  41 European Australians | Suppression | No comparison reported. |
| **Nauta et al. (2010)** | Cross Sectional  University Employees;  146 Chinese  180 Americans | Self-efficacy | No comparison reported. |
| **Nishiguchi et al. (2022)** | Cross Sectional  University Sample, General Community;  1200 Japanese  2200 Germans | Avoidance | Japanese reported significantly higher avoidance than Germans. |
| **O’Connor & Shimizu (2002)** | Cross Sectional  University Sample;  84 Japanese  82 British | Sense of Personal Control | Japanese reported significantly lower sense of personal control than British. |
| **Ogawa (2009)** | Cross Sectional  University Sample;  269 Japanese  256 Americans | Avoidance | Americans reported higher avoidance-related coping than Japanese.  Interdependent self-construal was positively correlated with avoidance-related coping.  Independent self-construal was negatively correlated with avoidance-related coping. |
| **Peng (1995)** | Cross Sectional  General Community;  132 Chinese Americans  339 Americans | Primary Control Appraisal  Secondary Control Appraisal | Chinese Americans scored higher on secondary control than Americans.  Americans scored higher on primary control than Chinese Americans. |
| **Perera & Chang (2015)** | Cross Sectional  University Sample;  123 East Asian Americans  117 European Americans | Avoidant Emotional Coping | No significant cultural group differences in avoidance. |
| **Schunk et al. (2022a)** | Cross Sectional  University Sample;  524 Japanese  476 Austrians/Germans | Expressive Suppression  Cognitive Reappraisal,  Rumination  Distraction  Savouring  Empathic suppression  Controlled expression  Uncontrolled expression  Acceptance | Japanese reported more distraction, rumination of negative emotions, and empathetic suppression of positive emotions.  Germans reported more reappraisal, acceptance, empathic suppression of negative emotions, and savouring of positive emotions. |
| **Schunk et al. (2021)** | Cross Sectional  University Sample;  125 HKC  127 Japanese  148 Germans | Expressive Suppression  Cognitive Reappraisal  Rumination | No comparison reported. |
| **Schunk et al. (2022b)** | Cross Sectional  University Sample;  136 HKC  123 Japanese  129 Germans | Expressive Suppression  Cognitive Reappraisal  Rumination | Germans reported greater cognitive reappraisal than HKC.  No significant group difference in rumination and expressive suppression.  **Harmony seeking** was   - Uncorrelated with rumination among Germans, correlated with less rumination among HKC, correlated with more rumination among Japanese. - Correlated with higher reappraisal across cultures. - Correlated with less suppression only among Germans.   **Rejection avoidance** was:   - Correlated with rumination across cultural groups, with this relationship being strongest for HKC. - Correlated with less reappraisal among Germans and HKC but unrelated among Japanese. - Correlated with more suppression among Germans only. |
| **Shaffer et al. (2000)** | Cross Sectional  Women University Sample;  103 Chinese  185 HKC  126 Americans | Positive/Confrontational C oping  Avoidance Coping | Chinese and HKC women reported higher use of avoidance coping than U.S. women. |
| **Shaw et al. (1997)** | Cross Sectional  Family Caregivers of Patients with Alzheimer's Disease;  110 Chinese  139 Americans | Behavioural Confronting | No significant cultural group differences in behavioural confronting. |
| **Shim et al. (2006)** | Cross Sectional  Women with Breast Cancer;  112 Japanese  106 Koreans  195 Germans | Cognitive C oping/R eappraisal  Avoidance C oping | No comparison reported. |
| **Smith et al. (2016)** | Cross Sectional  University Sample;  519 Chinese  688 Finnish/British | Anger Regulation  *(Anger In, Anger Out, Anger Control)* | No comparison reported. |
| **Soto et al. (2011)** | Cross Sectional  University Sample;  100 HKC  71 European Americans | Expressive S uppression  Cognitive R eappraisal | HKC reported using suppression more than European Americans.  No significant group differences in cognitive reappraisal. |
| **Taku et al. (2009)** | Cross Sectional  University Sample, General Community;  431 Japanese  224 Americans | Intrusive R umination  Deliberate R umination | No comparison reported. |
| **Toussaint et al. (2023)** | Cross Sectional  University Sample;  204 Koreans  297 Americans | Rumination | No significant group differences in rumination. |
| **Tse (2017)** | Cross Sectional  University Sample;  353 Chinese  491 Americans | Emotional Expression Coping  Emotional Processing (*active attempts to acknowledge, explore meanings, and understand one’s emotions*) | No significant cultural group differences in use of emotional processing and expression coping  For both groups:   - Independent and interdependent self-construal positively correlated with emotional expression coping. - Interdependent self-construal positively correlated with emotional processing.   Independent self-construal positively correlated with emotional processing for Americans but not Chinese. |
| **Turner (2022)** | Mixed-method Design  General Community;  122 Japanese  102 Americans  111 Danish | Narrative I dentity (*one’s internalised and evolving story of the self, functioning to provide life with some degree of meaning, purpose and temporal coherence*):  Agency  Meaning-M aking | Japanese had significantly lower meaning making than Americans and Danish.  No significant cultural group differences in agency appraisal.  Additional culturally-specific narrative themes: **Denmark:**   - Communal growth (*learning to accept and appreciate help and better help others*) - Balanced affect (*consider event outcome positive AND negative*) - Normality (*consider personal events as normal rather than unique or special*)   **Japan:**   - Acceptance (*fatalism, aversion to the idea of making meaning*) - Attribution of blame (*use of self-blame or blaming external forces*) - Open-ended (*leaving the event as open-ended as opposed to coming to a resolution*) |
| **Xiu et al. (2016)** | Cross Sectional  Bereaved Parents;  32 Chinese  33 Swiss | Sense of C oherence (*a metacognitive style of balancing the negative and positive sides of a stressful event, reflecting on a situation comprehensively and form various perspectives, and believing that experiences are manageable*)  Social Axioms  *(reward for application, social cynicism, social complexity, fate control, religiosity)* | Chinese scored higher in social complexity and lower in social cynicism.  No cultural group difference in sense of coherent, reward for application, fate control and religiosity. |
| **Yamaguchi et al. (2015)** | Cross Sectional  General Community;  1027 Japanese  1255 Americans | Anger R egulation  *(Anger In, Anger Out, Anger Control)* | No comparison reported. |
| **Yu et al. (2023)** | Cross Sectional  General Community;  205 Taiwanese  247 Americans | Positive E motional S uppression  Negative E motional S uppression | No comparison reported. |
| **Zaragoza Scherman et al. (2015)** | Cross Sectional  General Community;  154 Chinese  142 Danish | Event C entrality | Emotionally positive events were considered more central than negative events across cultures.  Chinese and Danish did not differ significantly in rating of both positive and negative event centrality. |

*Note.* HKC refers to Hong Kong Chinese. As the inclusion criteria required studies to directly compare the association between emotion regulation and mental health across cultural groups, studies that did not report levels or frequency of emotion regulation strategy use were still eligible (reported as “No comparison reported” in this table), provided they examined cross-cultural differences in the association between emotion regulation and mental health outcomes.

*Table 4.* Detailed findings on cultural differences in the associations between emotion regulation and mental health

| **Study** | **Mental Health Outcome/s** | **Key Findings** |
| --- | --- | --- |
| **Adams (2003**) | Psychiatric D istress  Depression | - The positive correlation between escape-avoidance coping and distress was significantly larger for Japanese Americans than for Anglo Americans. - None of the other appraisal or coping variables were significantly correlated with distress for both groups. |
| **Akutsu et al. (2016)** | Life Satisfaction | - The negative impact of anger-in on life satisfaction was stronger for Japanese than Americans. - The positive association between anger out/control and life satisfaction was significant only for Japanese and not Americans. |
| **Aldwin & Greenberger (1987)** | Depression | - Perceived social other's (parents) traditional value appraisals were associated with depression for Korean but not Caucasians. - For Caucasian students, self-appraisal was more important than social other's appraisals. - Adherence to modern values was associated with lower depression for Caucasian but not Korean students. - Coping strategies examined in this study were not associated with depression in Caucasian students. - Acceptance, problem solving, and expressing emotions were associated with more depression for Koreans only. |
| **Au et al., (2012)** | Self-esteem | - Negotiable fate was positively associated with self-esteem in Chinese but not Americans. - Fatalism was associated with lower self-esteem across both cultures. |
| **Bjorck et al., (2001)** | Depression  Anxiety | - Depression was positively correlated with accepting responsibility and escape-avoidance for both groups. - Anxiety was positively correlated with escape-avoidance for both groups. - Anxiety was positively correlated with accepting responsibility and loss appraisal for Koreans only. |
| **Bonanno et al. (2005)** | Distress | Culture moderated the relationship between T1 deliberate avoidance and T2 distress, such that deliberate avoidance only predicted more distress for Americans but not Chinese. |
| **Cai et al. (2007)** | Self-Esteem | - Self-esteem was significantly correlated with cognitive self-evaluations for both Chinese and Americans. - Cognitive self-evaluations did not mediate the relationship between culture and self-esteem. |
| **Chang & Yang (2024)** | Loneliness  Depressive S ymptoms Hopelessness | - For both American and Chinese adults, positive problem orientation was negatively correlated with loneliness, depressive symptoms and hopelessness. - For both American and Chinese adults, negative problem orientation and avoidance were positively correlated with loneliness, depressive symptoms and hopelessness. - Rational problem solving was negatively correlated with loneliness and hopelessness across Americans and Chinese. - Rational problem solving was positively associated with depression only for Chinese but not Americans. |
| **Chataway & Berry (1989**) | Acculturative S tress Physical and P sychological H ealth | - Detachment was positively correlated with stress for HKC only. - Self-blame was positively correlated with stress for French only. - Problem solving, wishful thinking, seeking social support, positive thinking, tension-reduction, and withdrawal were not correlated with stress across groups. |
| **Chen et al. (2020)** | Depressive Symptoms | - Higher suppression frequency was associated with more depressive symptoms for Americans but not Chinese. - Higher suppression ability was associated with fewer depressive symptoms across cultures. - For Chinese but not American, increased suppression frequency was associated with higher ability to suppress in contextually appropriate scenarios, which in turn makes the habitual use of suppression associated with fewer depressive symptoms. |
| **Choi & Miyamoto (2023)** | Depressive S ymptoms  Trait A nxiety S ymptoms | - The positive association between rumination and depressive symptoms was stronger among European Americans than East Asians. - No cultural differences in the link between rumination and anxiety symptoms. |
| **Chung et al. (2020)** | Existential Loneliness | - Both Chinese and Swedes associated existential loneliness with loss of control of life. - Both Chinese and Swedes coped with existential loneliness through acceptance, positive reframing (reappraisal) and self-reflection. - Chinese participants defined oneself through their family and their role within it, whereas the Swedes defined it through participating in friendship and leisure activities, achieving personal accomplishment, and meeting their individual needs. |
| **Corey & Allen (2013)** | Perceived Stress | Active coping was not associated with perceived stress in the path model across Chinese Americans and Caucasian Americans. |
| **Engelbrecht & Jobson (2014)** | Psychological A djustment *(PTSD Symptomatology)* | - Perceived control was negatively correlated with PTSD symptoms for British but not Asians. - Attentional activity was negatively associated with PTSD symptoms for Asians but not British. - PTCI total was positively associated with PTSD for British but not Asian. - PTCI negative self was positively associated with PTSD for both groups. - PTCI negative world was only positively associated with PTSD for British, but not Asian. - PTCI self-blame was not associated with PTSD for both groups. |
| **Gage et al. (2015)** | Depressive S ymptoms  Anxiety S ymptoms | - Across groups, overall self-consistency was not correlated with anxiety/depression symptoms. - Across groups, consistency to undesirable characteristics (either endorsing or denying undesirable characteristics) was important for depression and anxiety symptoms. - For British but not East Asian, consistently denying desirable characteristics correlated with anxiety. - No correlation between desirable characteristic endorsement and symptomology across groups. |
| **Haliczer et al. (2020)** | Borderline P ersonality D isorder F eatures  Borderline P ersonality D isorder D iagnosis | - There was a significant, positive relationship between Nonacceptance and Affective Instability among White but not East Asian participants. Race did not moderate any other relationship between difficulties in ER and BPD features. - There was a significant, positive relationship between Low Emotional Awareness and BPD diagnosis among White but not East Asian participants. |
| **Hamamura & Mearns (2019)** | Depressive Symptoms | - NMRE was negatively associated with depression for both Japanese and Americans. - Avoidant coping was positively associated with depression in Americans but not Japanese. |
| **He et al. (2021)** | Negative Emotion | ‘White’ participants experienced a more substantial reduction in negative emotion ratings when employing reappraisal compared to East Asian participants. |
| **Hirano & Ishii (2024)** | Happiness  Loneliness | - The indirect effect of culture on happiness through reappraisal, suppression, and avoidant coping were significant; Japanese participants reported lower happiness via greater suppression and avoidant coping, and less reappraisal. - The indirect effect of culture on loneliness via reappraisal, suppression, and avoidant coping were significant; Japanese participants reported greater loneliness via greater suppression and avoidant coping, and less reappraisal. |
| **Ji & Wang (2024)** | Affect | Self-continuity, but not lay theory of change, mediated the relationship between culture and affects; Chinese reported greater self-continuity, which in turn predicted greater positive current affect. |
| **Ji et al. (2022)** | Affect | MISE was associated with higher positive affect for both Chinese and Canadians.  MISE mediated cultural differences in affect; Chinese demonstrated greater tendency to see meaning in stressful experiences, which in turn was associated with higher positive affect. |
| **Jobson et al. (2024)** | PTSD S ymptoms | - Chinese Australians with greater Chinese cultural beliefs about adversity were atemporally associated with fewer PTSD symptoms - significant for low self-construal index, and medium self-construal index, but not high self-construal index - significant for those with medium holism, and high holism, but not low holism. - For Chinese Australian, fewer fatalism appraisals were associated with fewer PTSD symptoms - significant for those with medium self-construal index and high self-construal index, but not low self construal index - significant for those with medium holism, and high holism, but not low holism. - For Chinese Australians, lack of secondary control was associated with more PTSD symptoms. - significant regardless of self-construal and holism level. - For European Australians, lack of primary control appraisals was atemporally associated with more PTSD symptoms - significant regardless of self-construal and holism level. |
| **Kahn et al. (2017)** | Depressive Symptoms  Life Satisfaction | - Distress disclosure was associated with less depressive symptoms; the association was stronger for Taiwanese than Americans. - Distress disclosure was associated with higher life satisfaction; the association was stronger for Americans than Taiwanese. |
| **Kalibatseva & Leong (2018)** | Depressive Symptoms | - Cognitive reappraisal was negatively associated with depressive symptoms across Chinese Americans and European Americans. - Expressive suppression was positively associated with depressive symptoms across Chinese Americans and European Americans. |
| **Krause et al. (1991)** | Depressive S ymptoms | - Personal control was correlated with less depressed affect for Americans but not Japanese. - Personal control was correlated with less somatic symptoms for both groups. |
| **Kwon & Kim (2019)** | Life S atisfaction | Suppression was negatively linked to life satisfaction across HKC and Americans. |
| **Kwon et al. (2013)** | Depressive Symptoms | - Reflective pondering, brooding and anger suppression (but not suppression and reappraisal) positively associated with depressive symptoms across Koreans and Americans. - Anger suppression was more positively associated with depression for Americans. - Reappraisal was more strongly associated with less depressive symptoms for Koreans. |
| **Leung et al. (2011)** | Psychological Distress | Avoidance coping was associated with more psychological distress across HKC and Anglo-Australians. |
| **Liddell & Williams (2019)** | Subjective C hanges in A ffect  Physiological R esponding to N egative S timuli | - EA benefited more from interpersonal priming relative to the WE group in terms of physiological responding, but that this did not extend beyond the immediate prime exposure phase. - EA group was better at implementing both interpersonal and intrapersonal ER strategies to moderate physiological responses to negative cues. |
| **Morling et al. (2003)** | Distress | - At T1 cross-sectionally, acceptance was associated with less distress for both cultural groups. - T1 acceptance predicted less T2 distress among American women, but predicted more T2 distress among Japanese women. - Social assurance and personal control did not predict overtime distress across groups. |
| **Nagulendran & Jobson (2020)** | Depression  PTSD D iagnosis | - Caucasians with PTSD reported significantly greater expressive suppression, thought suppression, rumination, experiential avoidance and worry, and lower levels of reappraisal than Caucasians without PTSD. - For the Caucasian group, all of these emotion regulation strategies significantly correlated with PTSD symptoms. - East Asians with PTSD reported significantly greater experiential avoidance than East Asians without PTSD, reappraisal, thought suppression and general emotion dysregulation did not differentiate between East Asians with and without PTSD. - For the East Asian group worry and emotional expression did not correlate with PTSD symptoms. - The differences in expressive suppression and worry between East Asians with and without PTSD were less marked than the Caucasian group.   In both groups:   - Those with PTSD reported more habitual and trauma-rumination. - Habitual and trauma-rumination correlated with PTSD symptoms significantly. - Experiential avoidance and thought suppression also significantly correlated with PTSD symptoms in both groups, but the correlation coefficients were significantly weaker for the East Asian group when compared to the Caucasian group. |
| **Nagulendran et al. (2020)** | Biological M easures of E motional E xperiences  Positive and N egative A ffect  Trauma I ntrusion | - Across all groups, subjective negative affect increased and subjective positive affect reduced after watching the trauma film regardless of suppression use. - The East Asian suppression group had significantly increased parasympathetic (reflecting a decreased sense of threat) response while viewing the film relative to the East Asian control group, but no such difference was observed between the European Australian groups - suggesting suppression was specifically beneficial for the East Asian group during the film. - Regardless of cultural background, suppression predicted fewer intrusive memories immediately (5 min window) after viewing the film compared with the control group. - For the European Australian group, change in heart rate (recovery–during film) interacted with group (suppression vs control) in predicting weekly intrusions. However, this was not observed for the East Asian group. |
| **Nauta et al. (2010)** | Psychological S trains | Self-efficacy was significantly associated with lower psychological strains across Chinese and American employees. |
| **Nishiguchi et al. (2022)** | Obsessive-C ompulsive S ymptoms  Depressive S ymptoms  Paranoid I deation | Avoidance was:   - positively correlated with depressive and paranoia symptoms across groups. - positively correlated with Obsessive-compulsive symptoms across groups, but more strongly for Germans. |
| **O’Connor & Shimizu (2002)** | Perceived Stress  Psychological Distress | Sense of personal control was correlated with less perceived stress and psychological distress in the British sample but not correlated with any outcome among Japanese. |
| **Ogawa (2009)** | Stress | Stress level correlates positively with avoidance-related coping for American but not Japanese. |
| **Peng (1995)** | Wellbeing  Depression  Present Life Satisfaction Expected Future Life Satisfaction | - Primary control was associated with all wellbeing constructs for American and all but self-acceptance for Chinese American. - Primary control was associated with higher life satisfaction and lower depression for Americans but not associated for Chinese Americans. |
| **Perera & Chang (2015)** | Depressive Symptoms | Avoidant emotional coping with both academic and interpersonal stressful events was significantly associated with higher depressive symptoms for both groups. |
| **Schunk et al. (2022a)** | Subjective W ell-being  Depressive Symptoms | **Suppression**   - **Expressive suppression was negatively** associated with SWB and positively associated with depression for Germans but not Japanese. - For Japanese, **empathic suppression** was positively associated with subjective well-being and negatively associated with depression. - For Germans, empathic suppression was negatively associated with subjective well-being and positively associated with depression. - For Japanese, higher interdependent self-construal was associated with greater use of empathic suppression of negative emotions, which in turn was linked to higher subjective well-being and lower depression. - For Germans, higher interdependent self-construal was associated with greater use of expressive suppression, which in turn was linked to lower subjective well-being and higher depression.   **Reappraisal**   - For both cultures, greater **reappraisal of negative emotions** was associated with higher SWB and lower depression, but these associations were stronger among Germans.   **Acceptance**   - Although non-significant within each group, **acceptance** was positively associated with SWB for Japanese, and negatively associated for Germans.   **Rumination**   - **Rumination** was negatively associated with subjective well-being and positively associated with depression in both Japanese and Germans, but these associations were stronger among Germans.   **Distraction**   - Negative association between **distraction from positive emotions** and SWB only among Germans.   **Expression**   - **Uncontrolled expression of negative emotions** was positively associated with depression in both Japanese and Germans, but the association was stronger for Japanese. - **Uncontrolled expression of positive emotions** was positively associated with depression for the Japanese group, but negatively associated for the German group.   **Savoring**   - **Savoring** was negatively associated with depression only for Germans. |
| **Schunk et al. (2021)** | Life S atisfaction | - Suppression only negatively associated with life satisfaction among Germans. - Reappraisal positively associated with life satisfaction among Germans and HKC. - Rumination negatively associated with life satisfaction among Germans, HKC and Japanese. |
| **Schunk et al. (2022b)** | Life S atisfaction | - Across cultures, rumination was associated with lower life satisfaction. - Higher reappraisal was associated with higher life satisfaction among Germans and Japanese, but lower life satisfaction among HKC. The positive effect of reappraisal on life satisfaction was stronger among Japanese than Germans. - Suppression was associated with lower life satisfaction only among Germans. - **Harmony Seeking**: Among Japanese, harmony seeking was related to higher life satisfaction through greater reappraisal; Among Germans, harmony seeking was related to higher life satisfaction through increased reappraisal, and less suppression. - Reappraisal mediated a positive link between harmony seeking and life satisfaction for Germans and Japanese, but not HKC. - **Rejection Avoidance**: Among Japanese, rejection avoidance was related to lower life satisfaction through increased rumination; Among Germans, rejection avoidance was related to lower life satisfaction through more rumination, less reappraisal, and more suppression. |
| **Shaffer et al. (2000)** | Life Stress | Positive/Confrontational coping and avoidance coping were not associated with life stress across cultural groups. |
| **Shaw et al. (1997)** | Depression  Anxiety | Behavioural confronting was positively associated with depression and anxiety among Americans, but negatively associated with depression and not associated with anxiety among Chinese. |
| **Shim et al. (2006)** | Health R elated Q uality of L ife (mental health component) | Cognitive coping/appraisal was positively associated with mental health-related quality of life among Germans only, but not among Japanese and Koreans.  Avoidance coping was not associated with mental health-related quality of life across cultures. |
| **Smith et al. (2016**) | Depressive S ymptoms  Life S atisfaction | - Anger-in was positively associated with depression across both groups. - Anger-in was negatively associated with life satisfaction across both groups but stronger association for westerners. - Anger-control was associated with less depression and more life satisfaction for the Western group and not associated with either outcome for East Asians. - Anger-out was not associated with depression and life satisfaction across both groups. |
| **Soto et al. (2011)** | Life S atisfaction (psychological well-being)  Depressive S ymptomatology (psychological distress) | - Suppression was associated with higher depressed mood and lower life satisfaction for European Americans, but was unrelated for HKC. - Culture did not moderate the relationship between reappraisal and depressed mood or life satisfaction. |
| **Taku et al. (2009)** | Posttraumatic G rowth (Posttraumatic Growth Inventory) | - Intrusive rumination soon after the event was positively related to PTG but recent deliberate rumination most strongly predicted the current PTG for both samples. - For Americans, deliberate rumination recently was more important than deliberate rumination soon after the traumatic event, whereas for Japanese, deliberate rumination both soon after and recently were positively related to PTG. |
| **Toussaint et al. (2023)** | Depression | Rumination was correlated with depression in both cultures, but more strongly among Americans. |
| **Tse (2017)** | Somatic S ymptoms of D epression | - Emotional expression coping was associated with higher somatic symptoms for Chinese but not Americans. - Emotional processing was not associated with somatic symptoms for both cultural groups. |
| **Turner (2022)** | Depressive S ymptoms  Psychological Well-being  Life S atisfaction | - For all participants, wellbeing positively associated with the presence of redemption, agency and communion, and showed no relationship with contamination and meaning making. |
| **Xiu et al. (2016)** | Prolonged Grief Disorder Symptoms | - Grief severity of Swiss parents was influenced by intrapersonal factors (i.e., sense of coherence), whereas Chinese parents’ symptoms could be better predicted by culture-related world view. - Sense of coherence explained significant portions of the variance in Prolonged Grief Disorder symptoms in the Swiss sample but not in the Chinese group. - Social cynicism (but not other social axiom constructs) contributed to higher PG symptoms and only in the Chinese sample. - Fate control was unrelated to grief symptoms for both groups. |
| **Yamaguchi et al. (2015)** | Perceived S tress Social A nxiety | - Anger suppression (AI) positively influenced social anxiety and perceived stress status for both Americans and Japanese. - Outward anger expression (AO) negatively associated with social anxiety and positively with perceived stress status for Americans, but not associated with either outcome for Japanese. - Controlled anger expression (AC) negatively associated with social anxiety for both groups. - AC was negatively associated with perceived stress status for Americans but had no effect for Japanese. |
| **Yu et al. (2023)** | Life S atisfaction  Psychological W ellbeing  Depression S ymptoms | Positive emotions:   - Suppression of positive emotions was negatively associated with satisfaction with life, psychological well-being and positively associated with depression across cultures.   Negative emotions:   - Suppression of negative emotions was not associated with life satisfaction nor depression across groups. - Suppression of negative emotions was positively associated with psychological well-being for Americans but not Taiwanese. |
| **Zaragoza Scherman et al. (2015)** | Depressive S ymptoms PTSD S ymptoms  Life S atisfaction | - Across cultures, individuals with higher PTSD and depressive symptoms rated negative events as more central to their identity; whereas positive event centrality did not interact significantly with PTSD and depressive symptoms. - Life satisfaction was not significantly associated with the centrality of either event centrality type across Chinese and Danish participants. |

*Note.* HKC refers to Hong Kong Chinese.
